# Supplementary material for: Integrated multi-omics reveal polycomb repressive complex 2 restricts human trophoblast induction
Source: Nat Cell Biol. 2022 Jun 13;24(6):858–71. doi: 10.1038/s41556-022-00932-w (PMC9203278; doi:10.1038/s41556-022-00932-w)
Supplement: Supplementary file 1 — Supplementary Fig. 1. Flow cytometry and gating strategies for Extended Data Figs. 4i, 5d,i and 7b. [file 41556_2022_932_MOESM1_ESM.pdf]

nature portfolio

https://doi.org/10.1038/s41556-022-00932-w

Supplementary information

Integrated multi-omics reveal polycomb repressive complex 2 restricts human trophoblast induction

In the format provided by the authors and unedited

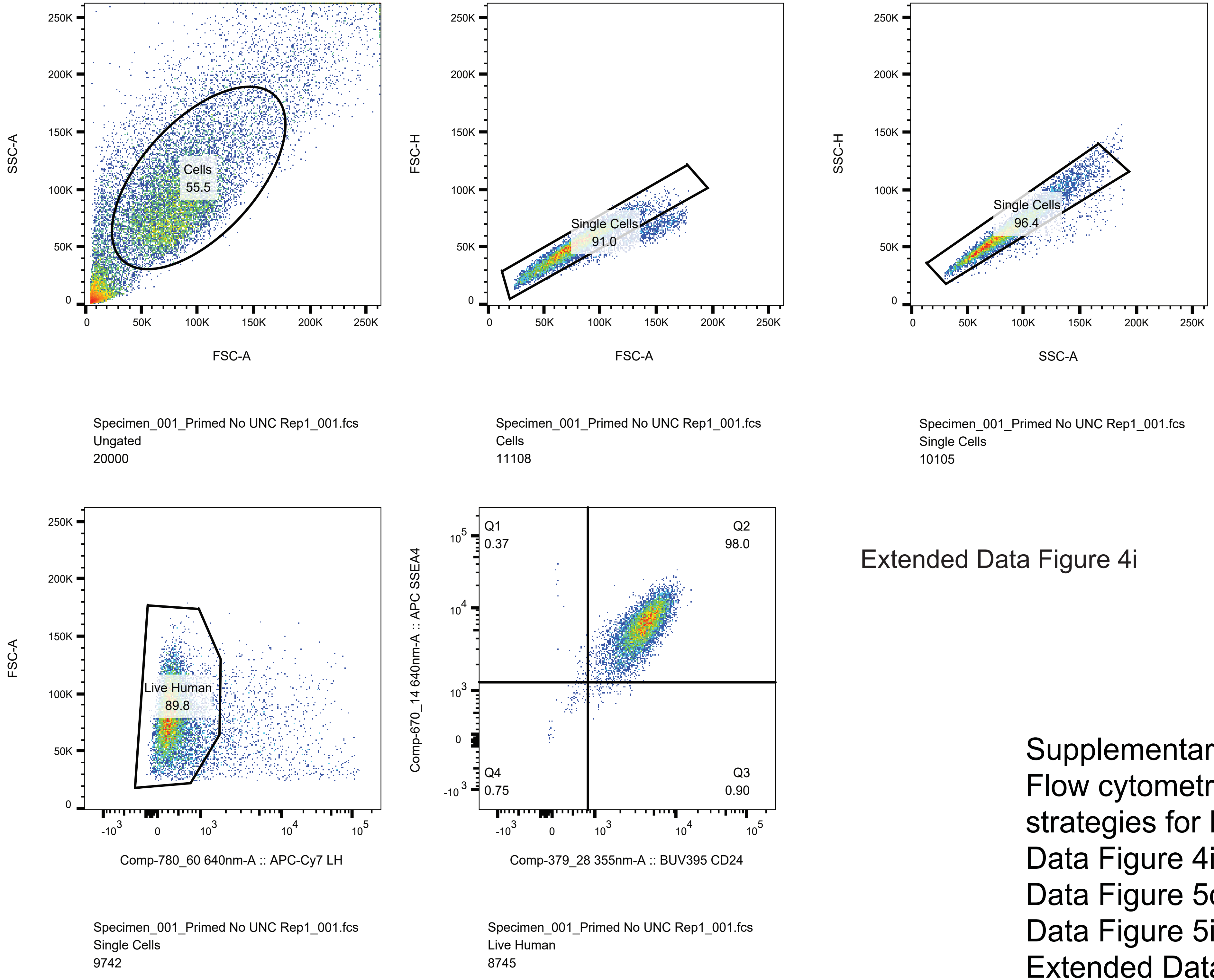

Extended Data Figure 4i

Supplementary Figure 1. Flow cytometry and gating strategies for Extended Data Figure 4i, Extended Data Figure 5d, Extended Data Figure 5i and Extended Data Figure 7b.

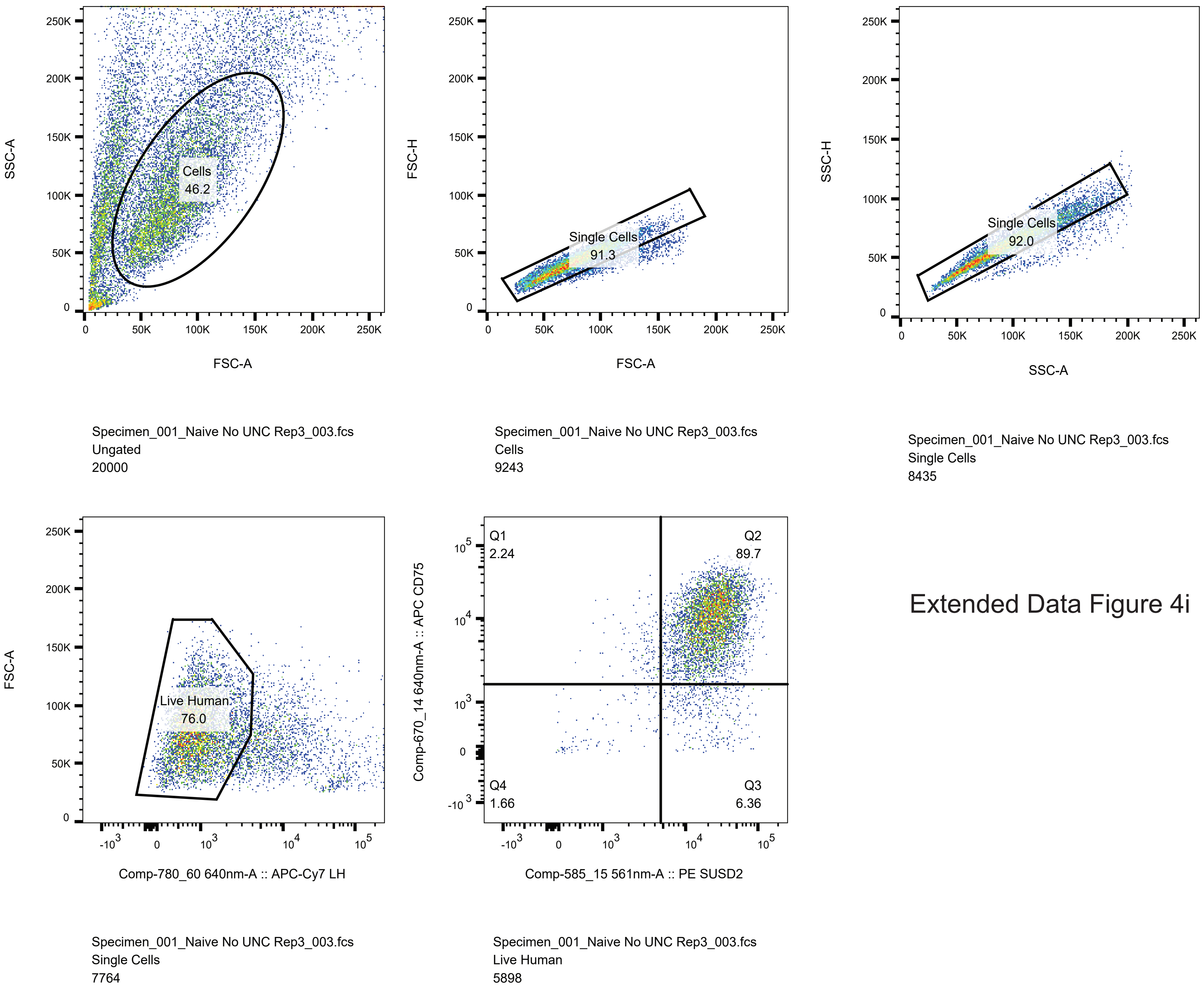

Extended Data Figure 4i

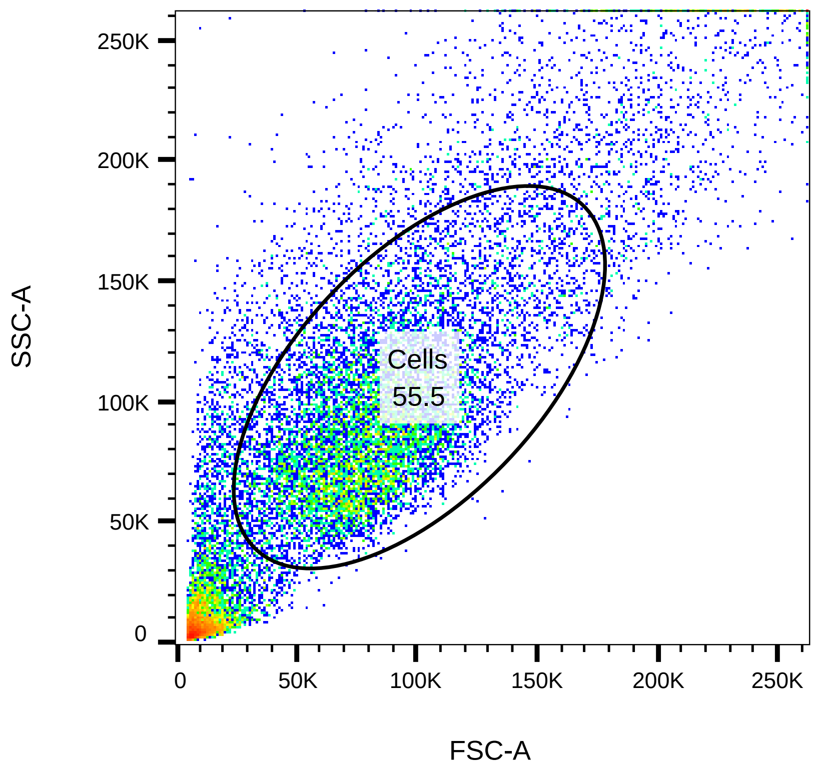

Specimen_001_Primed No UNC Rep1_001.fcs
Ungated
20000

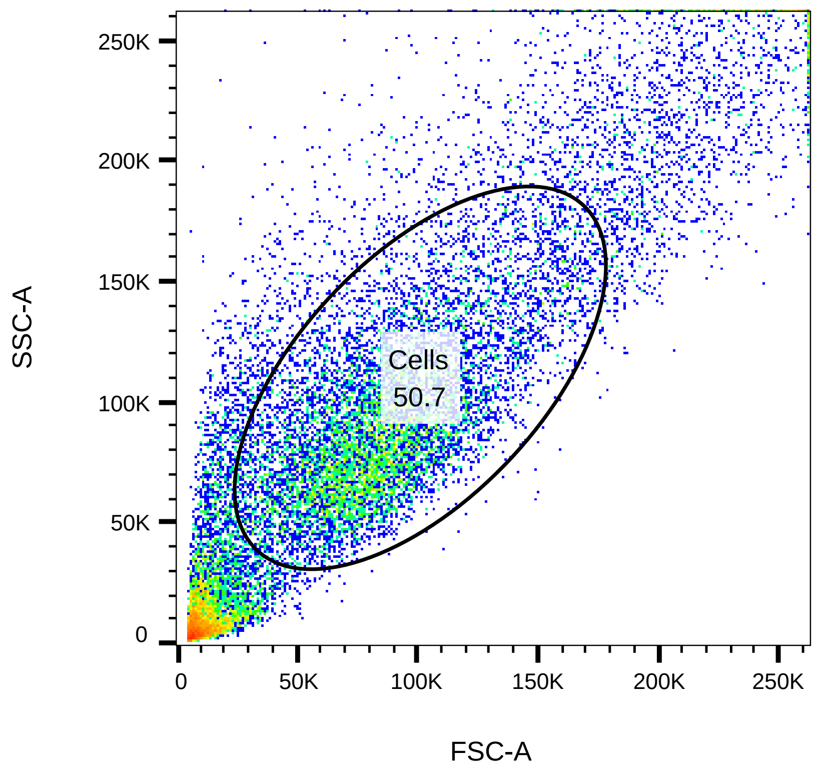

Specimen_001_Primed Plus UNC Rep1_002.fcs
Ungated
20000

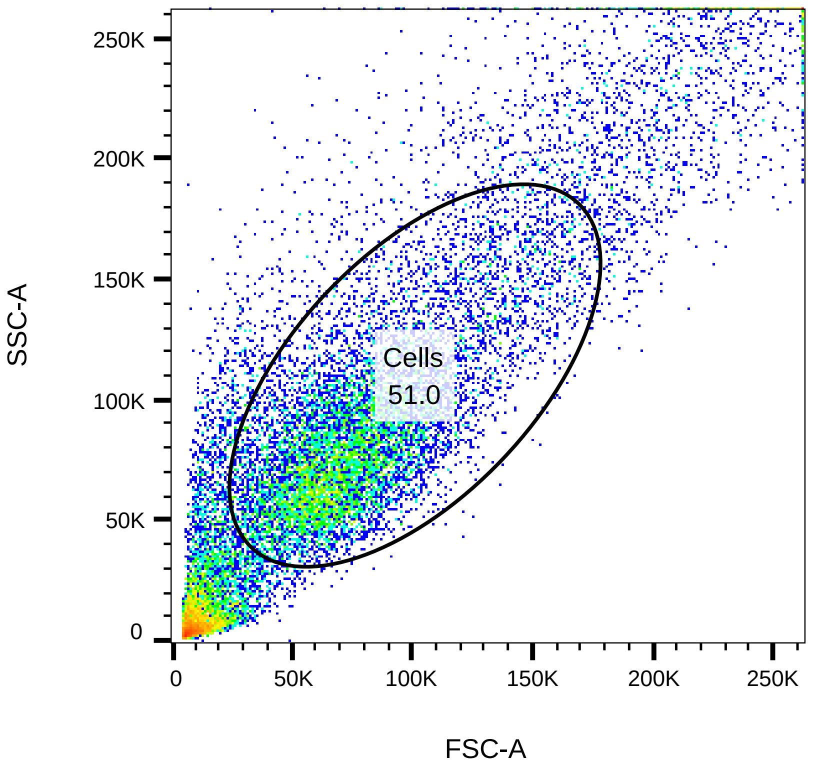

Specimen_001_Primed No UNC Rep2_003.fcs
Ungated
20000

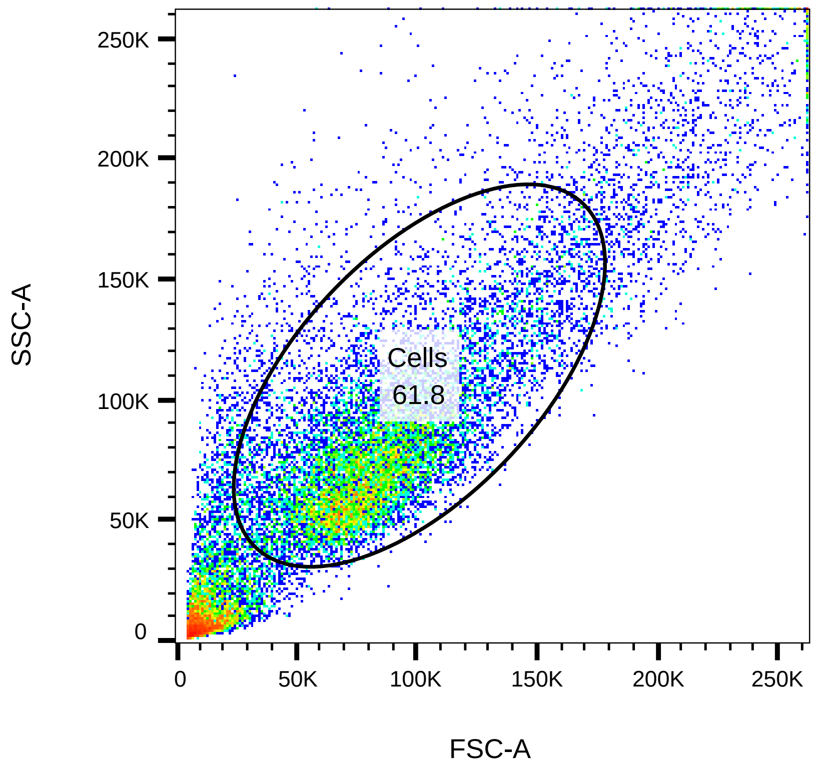

Specimen_001_Primed Plus UNC Rep2_004.fcs
Ungated
20000

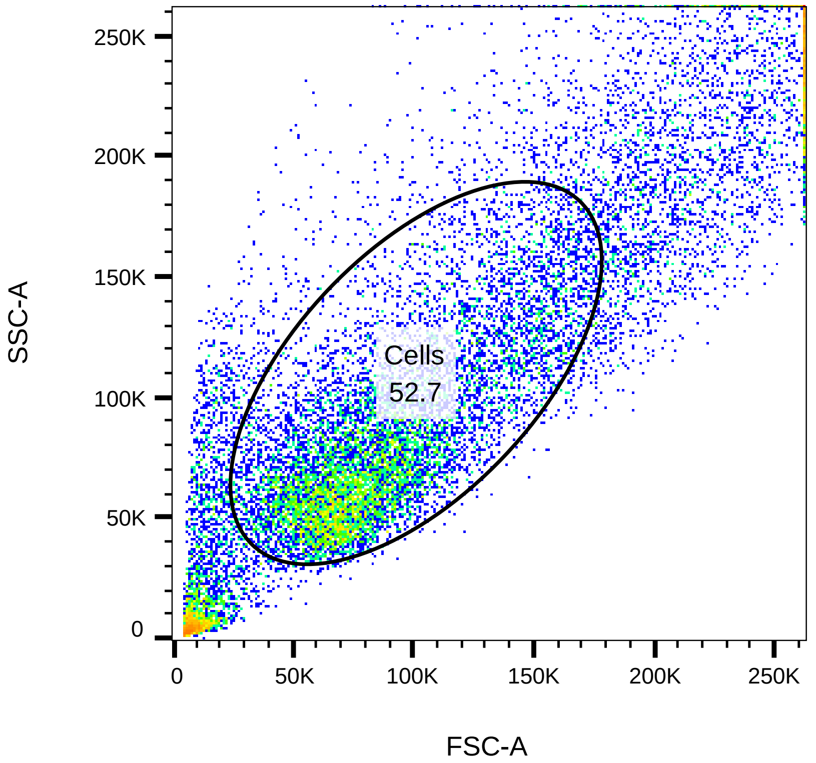

Specimen_001_Primed Plus No UNC Rep3_005.fcs
Ungated
20000

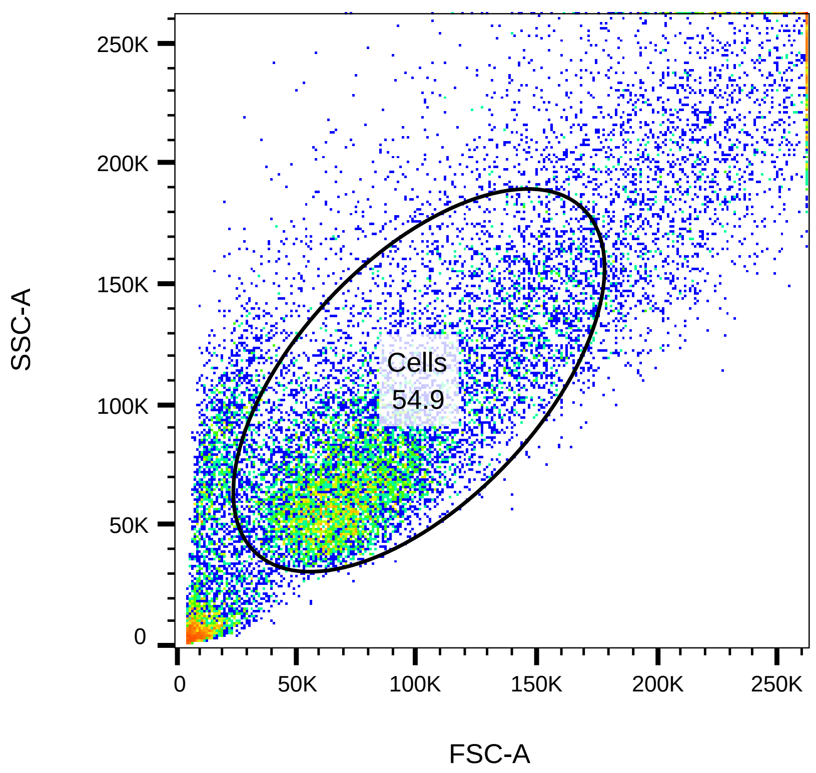

Specimen_001_Primed Plus UNC Rep3_006.fcs
Ungated
20000

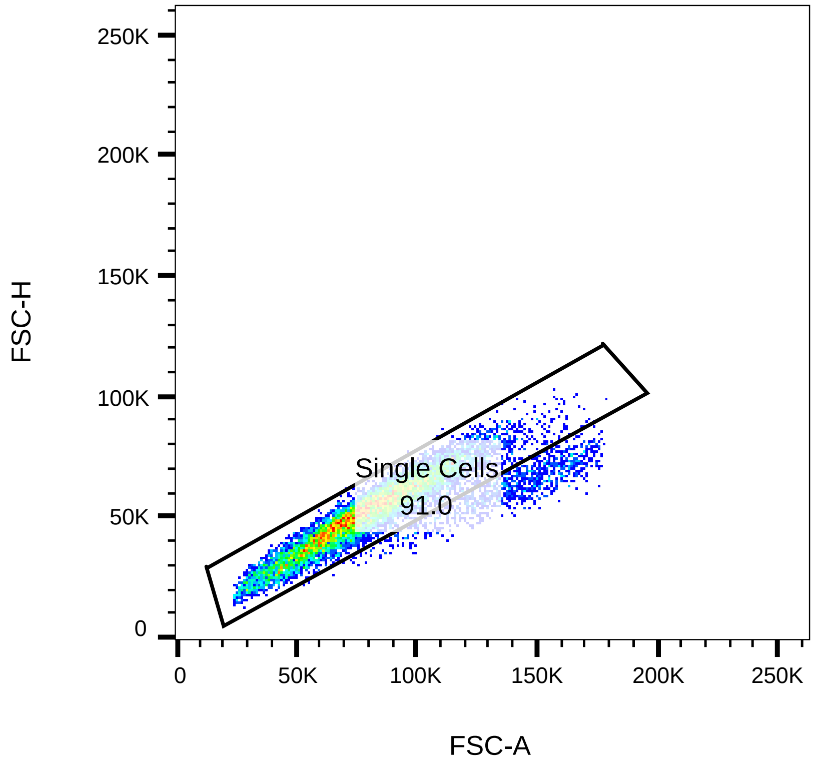

Specimen_001_Primed No UNC Rep1_001.fcs
Cells
11108

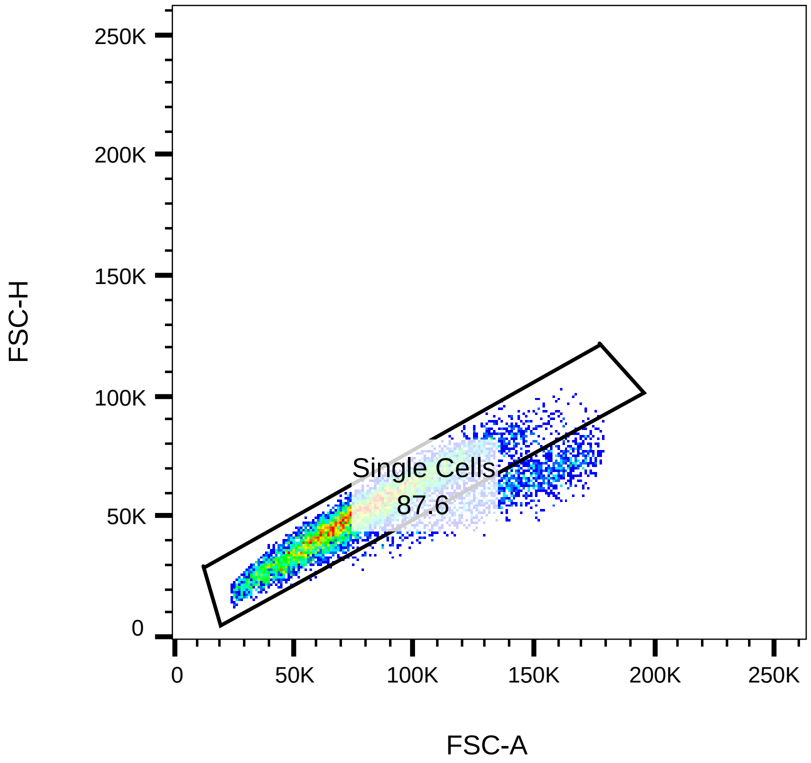

Specimen_001_Primed Plus UNC Rep1_002.fcs
Cells
10149

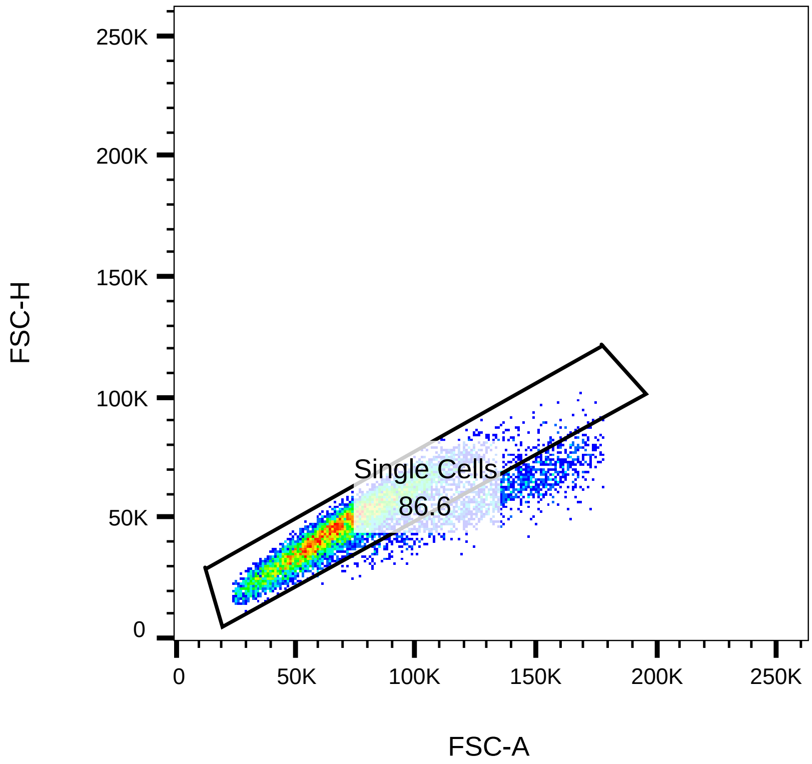

Specimen_001_Primed No UNC Rep2_003.fcs
Cells
10194

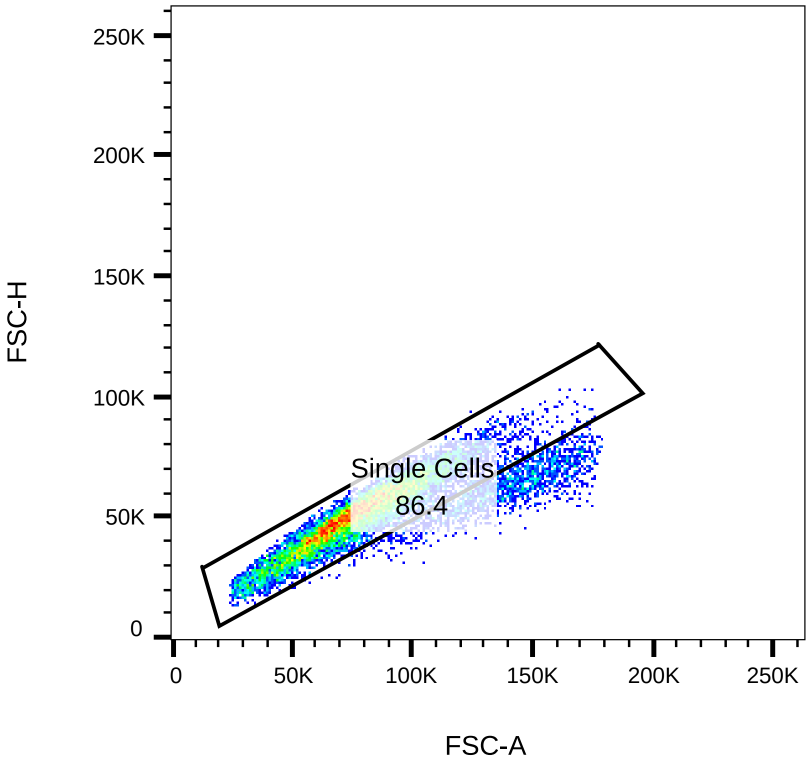

Specimen_001_Primed Plus UNC Rep2_004.fcs
Cells
12350

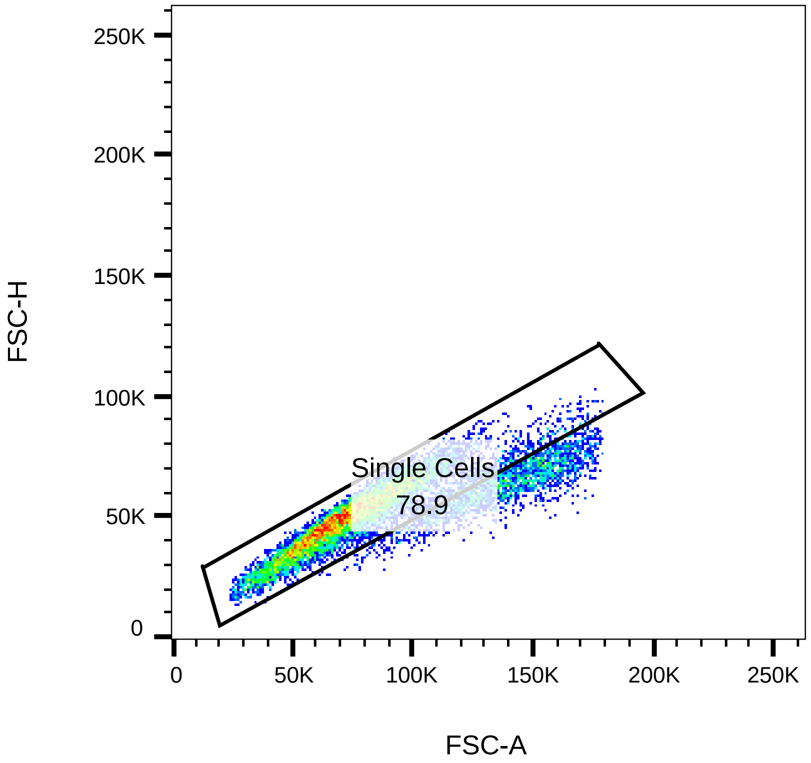

Specimen_001_Primed Plus No UNC Rep3_005.fcs
Cells
10544

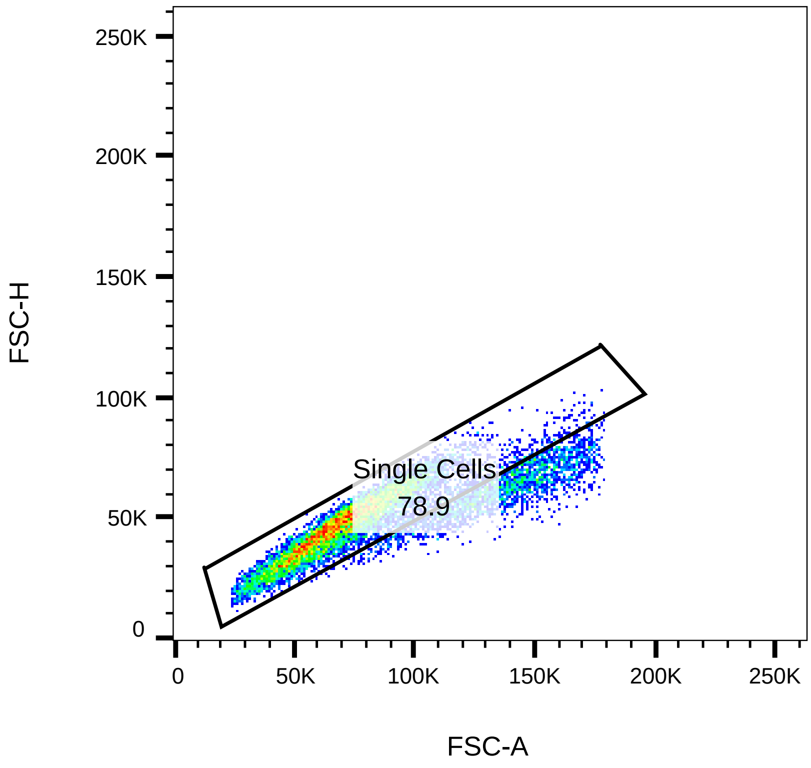

Specimen_001_Primed Plus UNC Rep3_006.fcs
Cells
10982

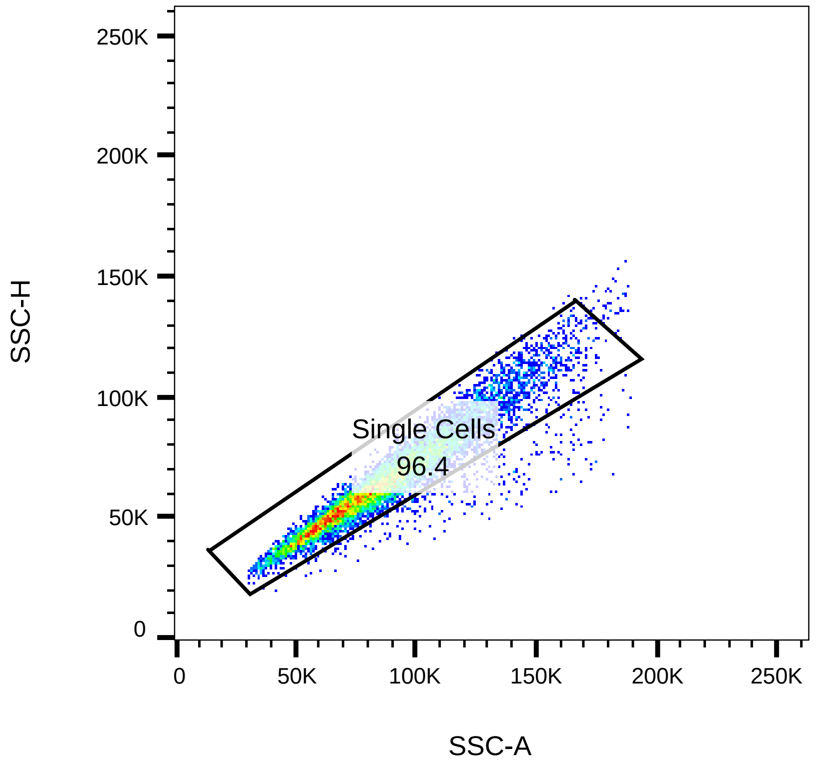

Specimen_001_Primed No UNC Rep1_001.fcs
Single Cells
10105

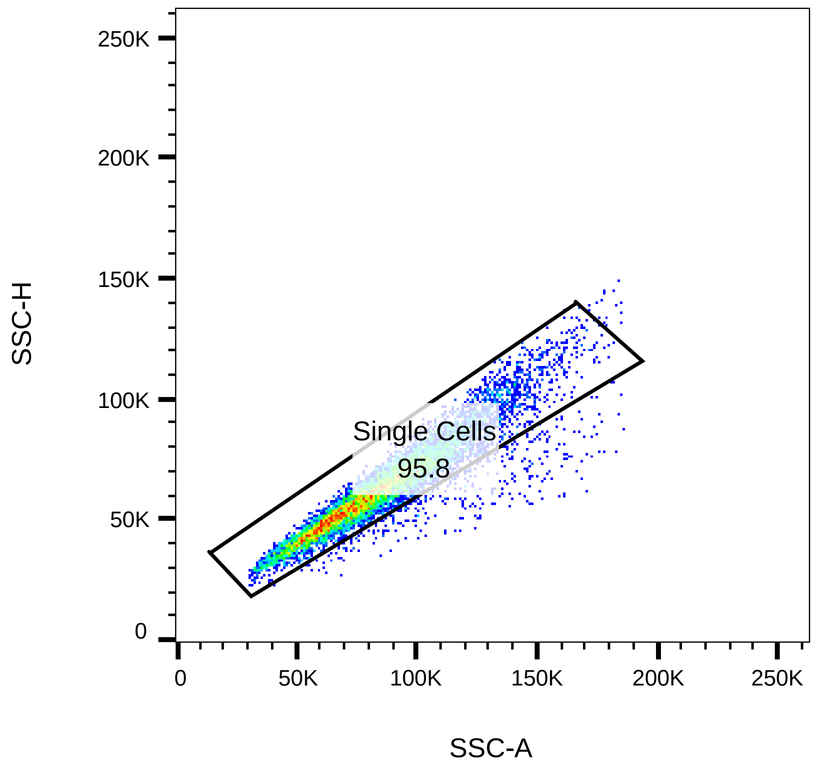

Specimen_001_Primed Plus UNC Rep1_002.fcs
Single Cells
8891

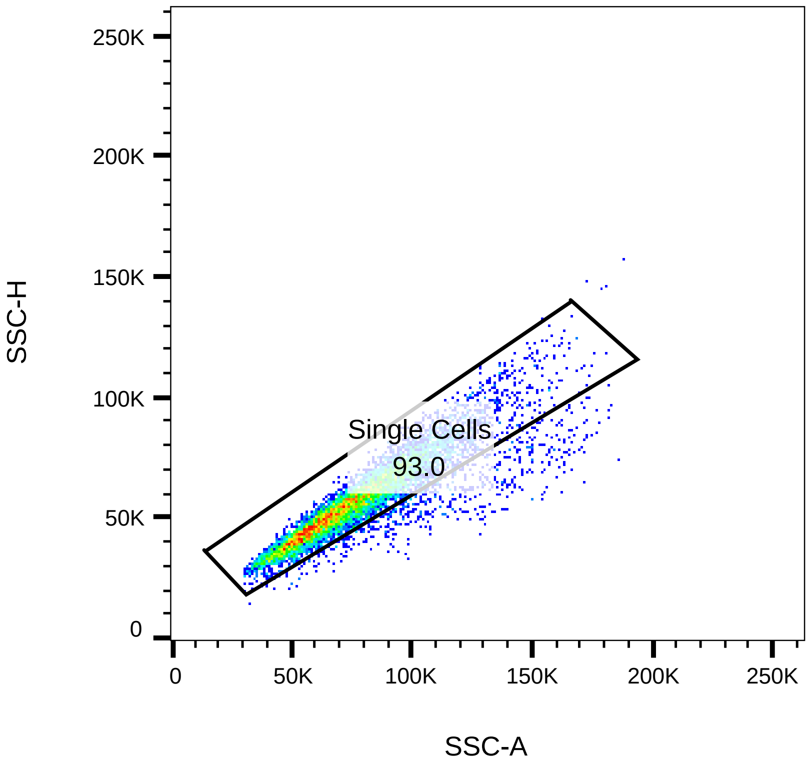

Specimen_001_Primed No UNC Rep2_003.fcs
Single Cells
8829

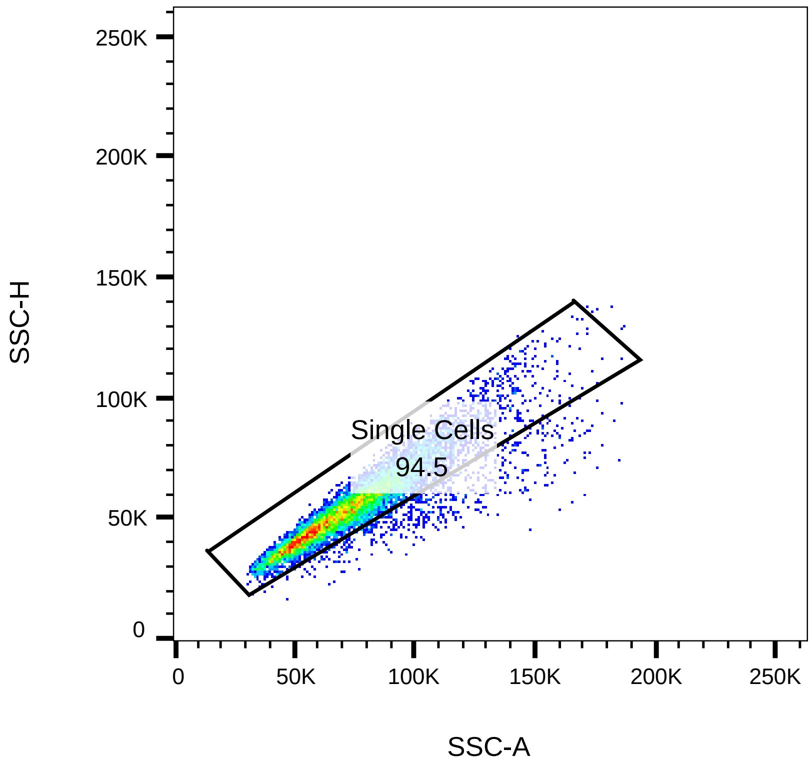

Specimen_001_Primed Plus UNC Rep2_004.fcs
Single Cells
10672

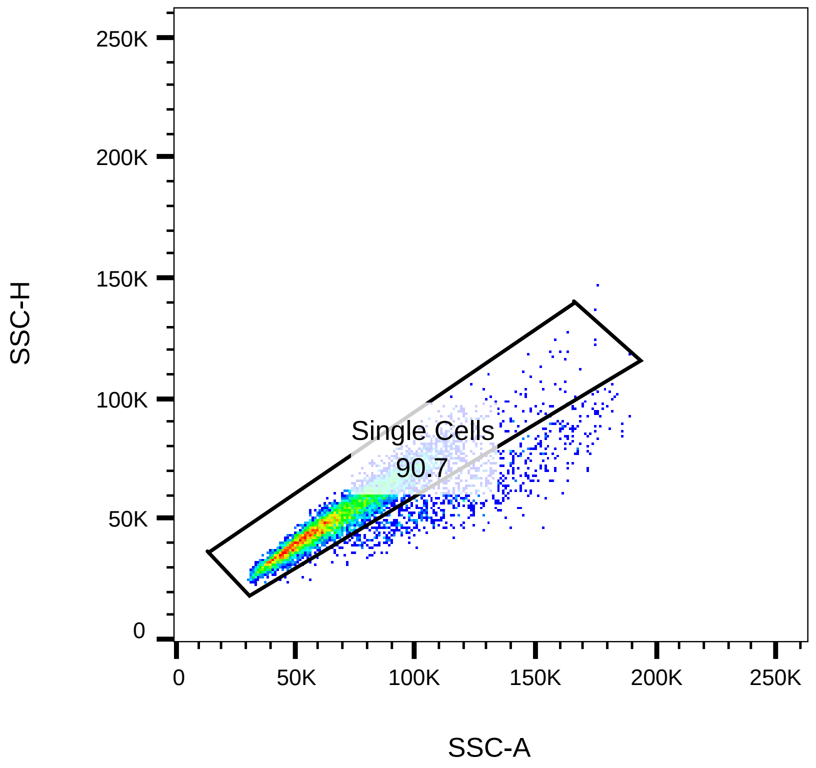

Specimen_001_Primed Plus No UNC Rep3_005.fcs
Single Cells
8315

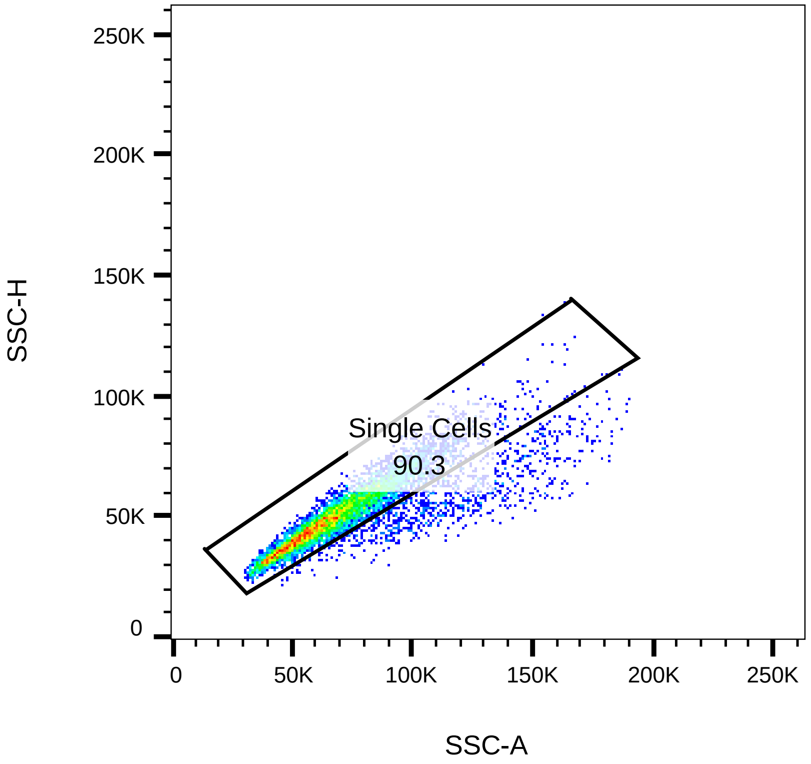

Specimen_001_Primed Plus UNC Rep3_006.fcs
Single Cells
8670

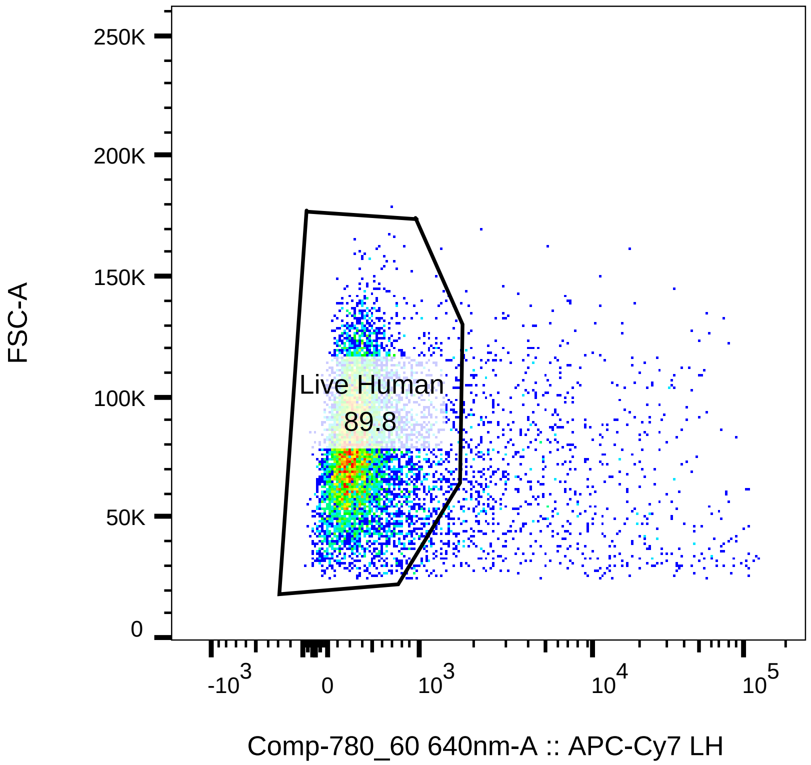

Specimen_001_Primed No UNC Rep1_001.fcs
Single Cells
9742

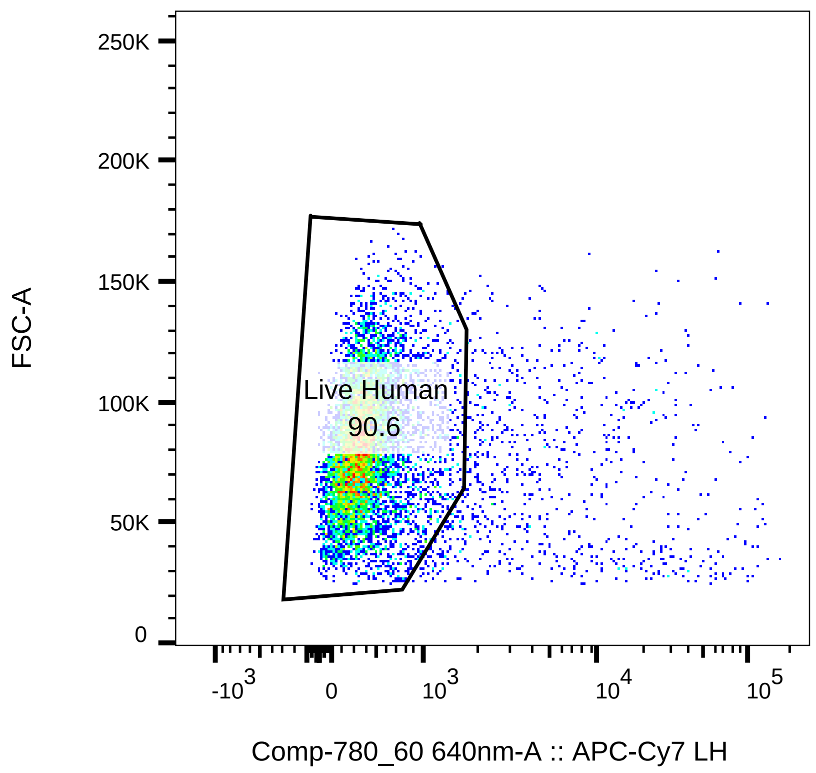

Specimen_001_Primed Plus UNC Rep1_002.fcs
Single Cells
8514

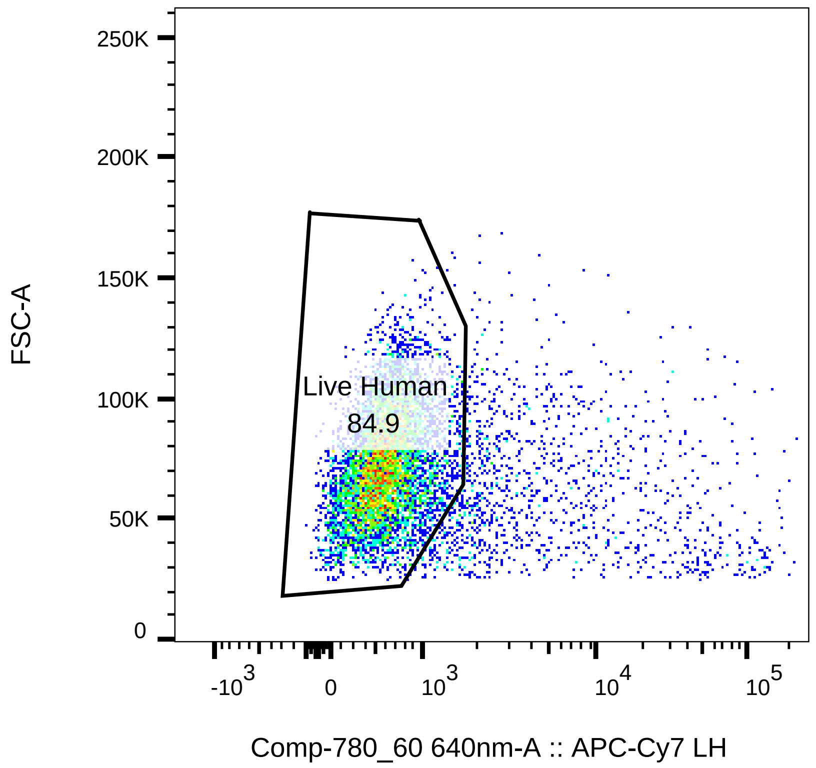

Specimen_001_Primed No UNC Rep2_003.fcs
Single Cells
8213

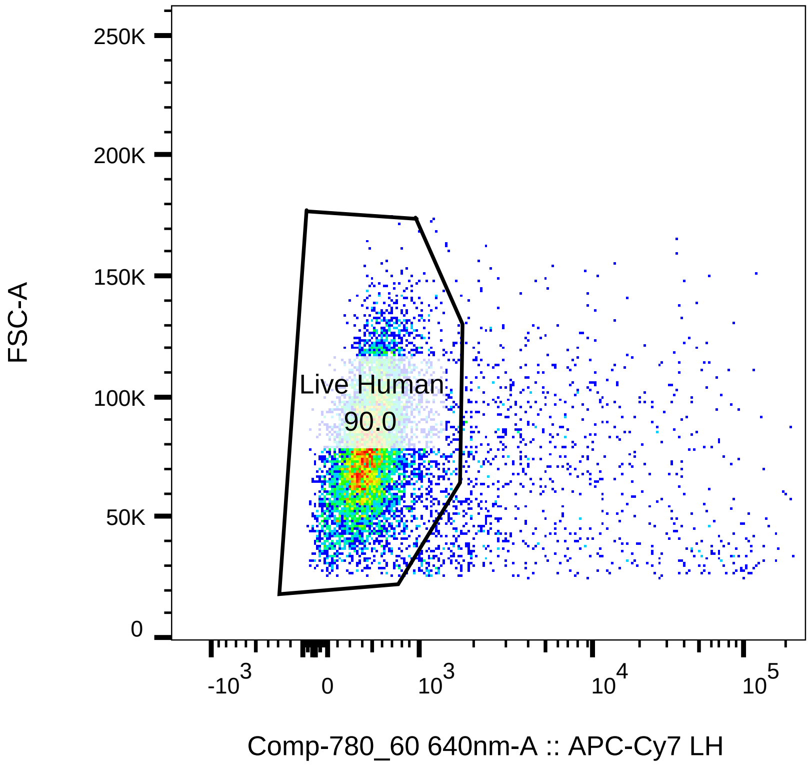

Specimen_001_Primed Plus UNC Rep2_004.fcs
Single Cells
10085

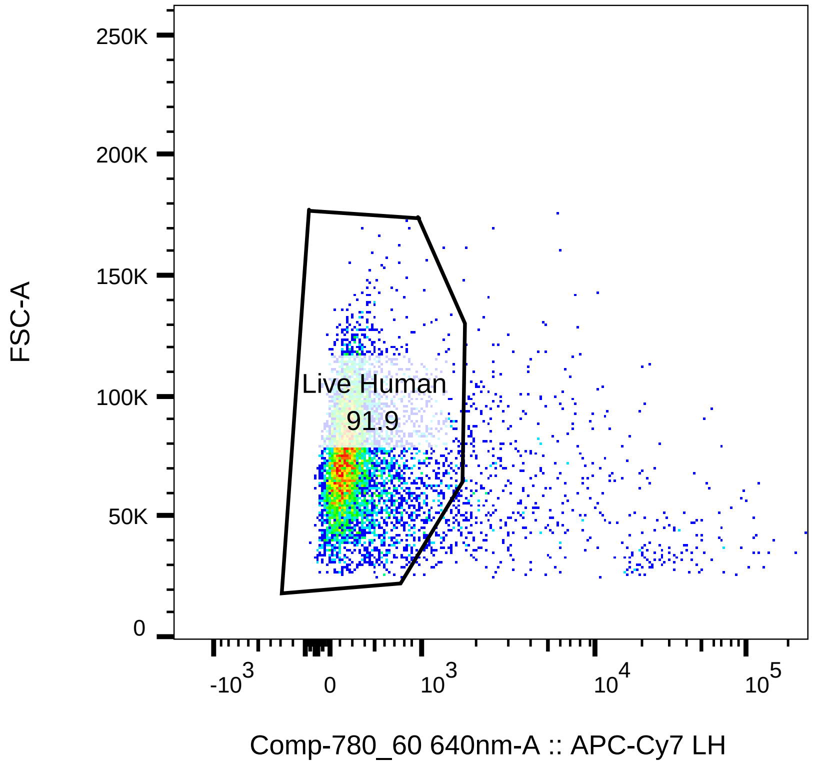

Specimen_001_Primed Plus No UNC Rep3_005.fcs
Single Cells
7538

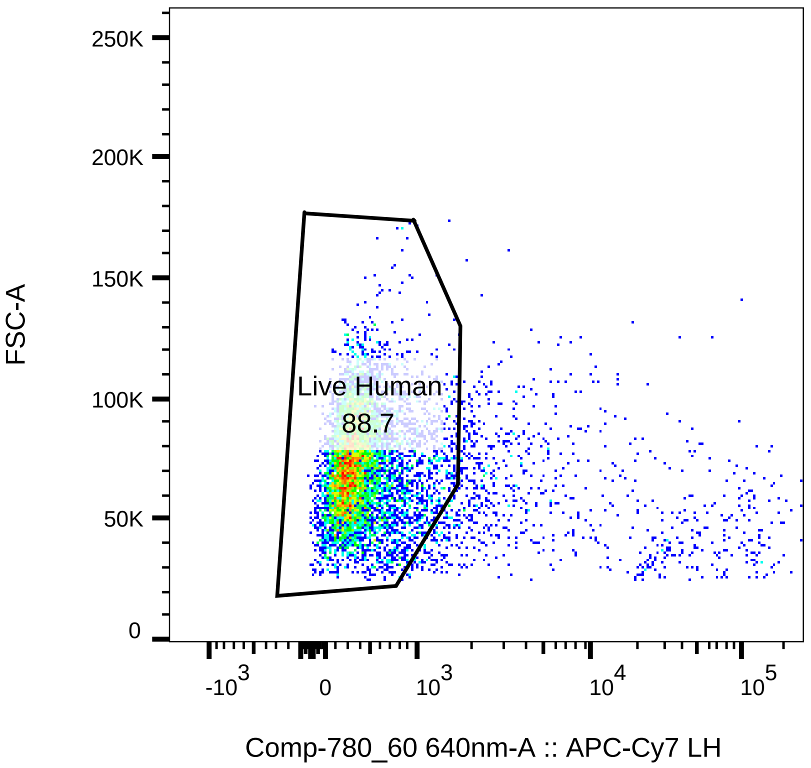

Specimen_001_Primed Plus UNC Rep3_006.fcs
Single Cells
7827

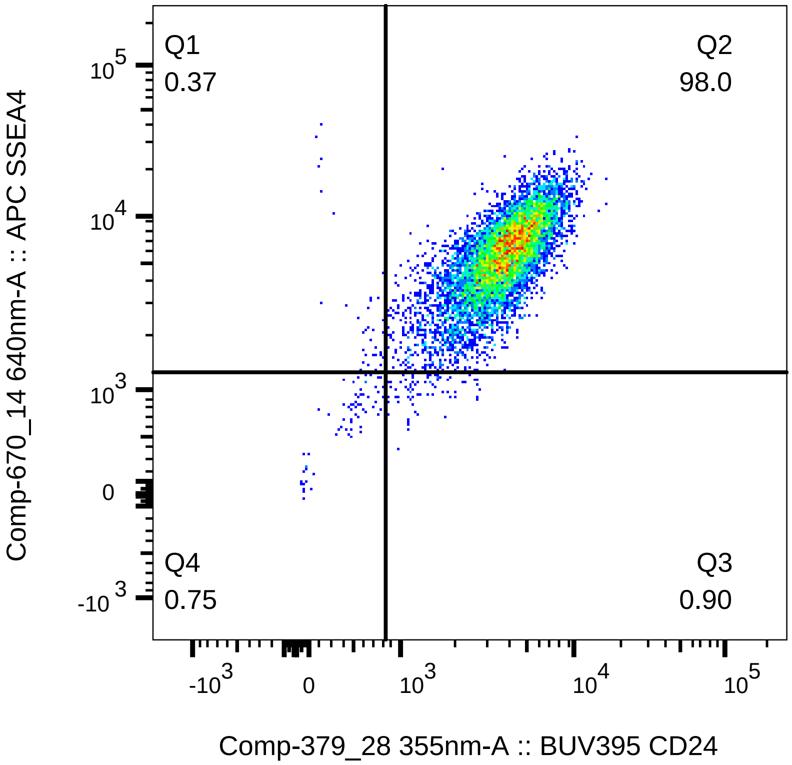

Specimen_001_Primed No UNC Rep1_001.fcs
Live Human
8745

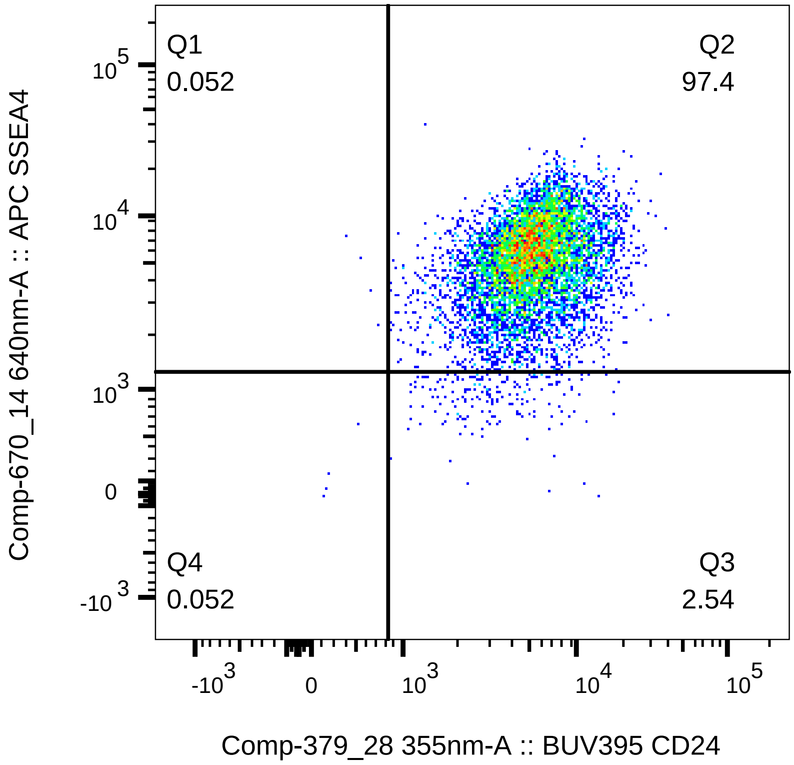

Specimen_001_Primed Plus UNC Rep1_002.fcs
Live Human
7711

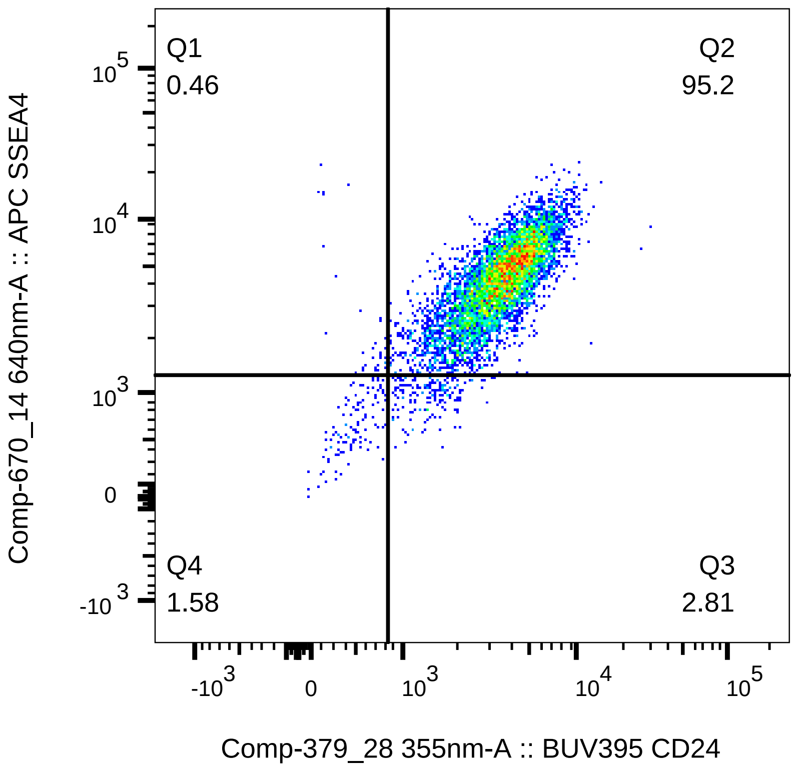

Specimen_001_Primed No UNC Rep2_003.fcs
Live Human
6974

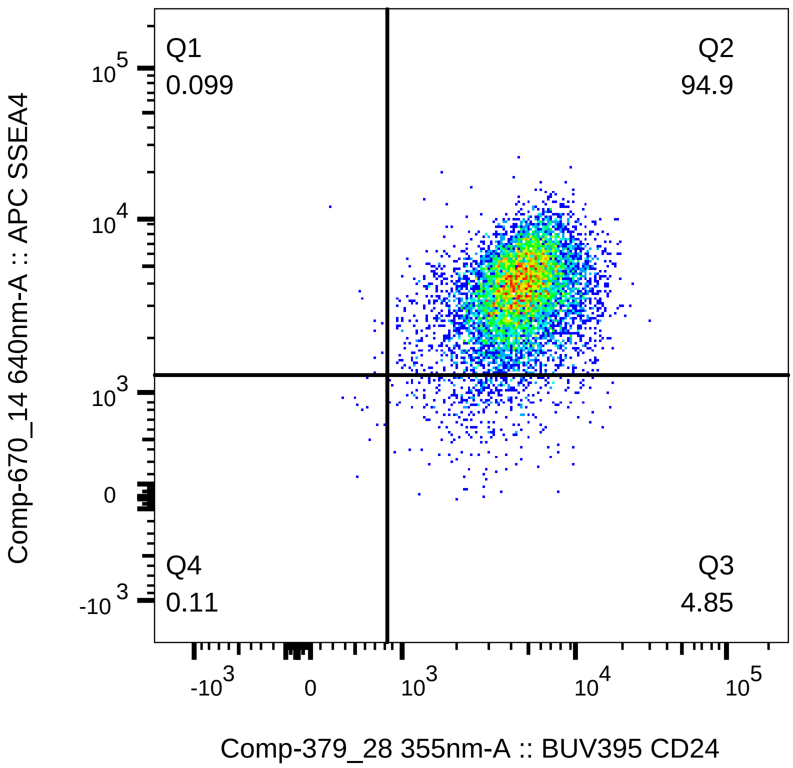

Specimen_001_Primed Plus UNC Rep2_004.fcs
Live Human
9077

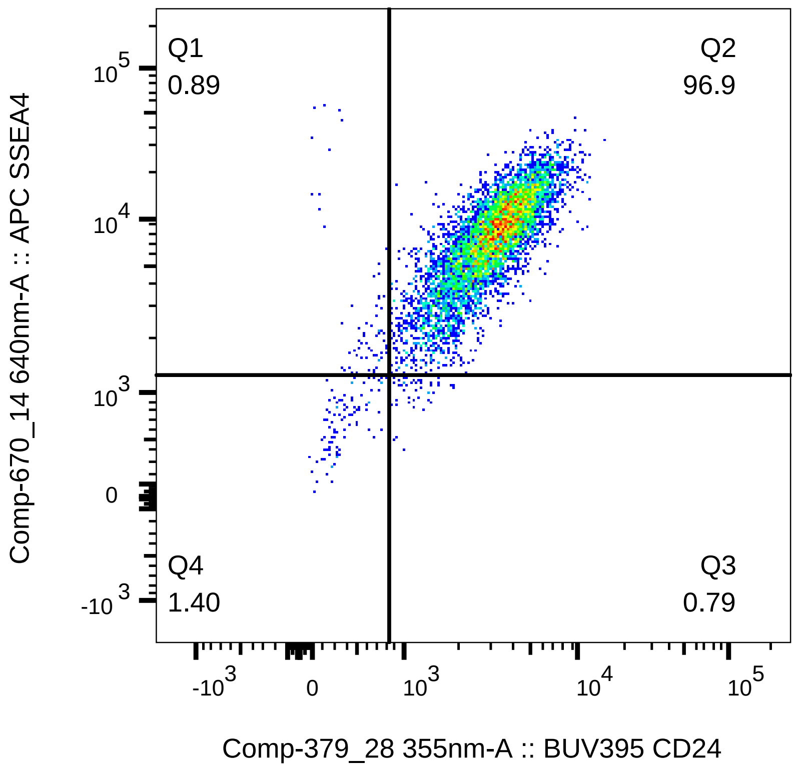

Specimen_001_Primed Plus No UNC Rep3_005.fcs
Live Human
6930

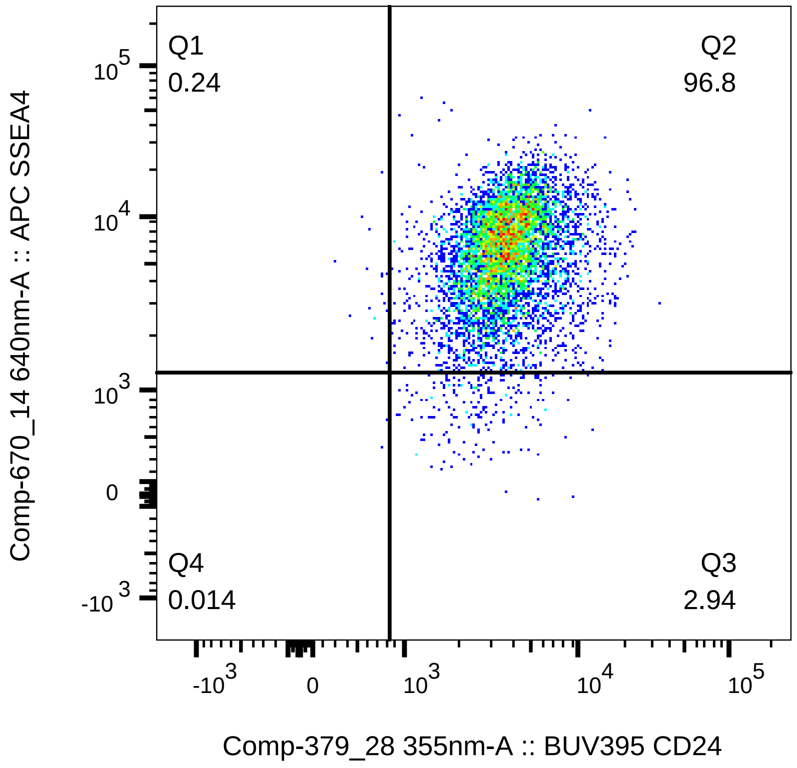

Specimen_001_Primed Plus UNC Rep3_006.fcs
Live Human
6941

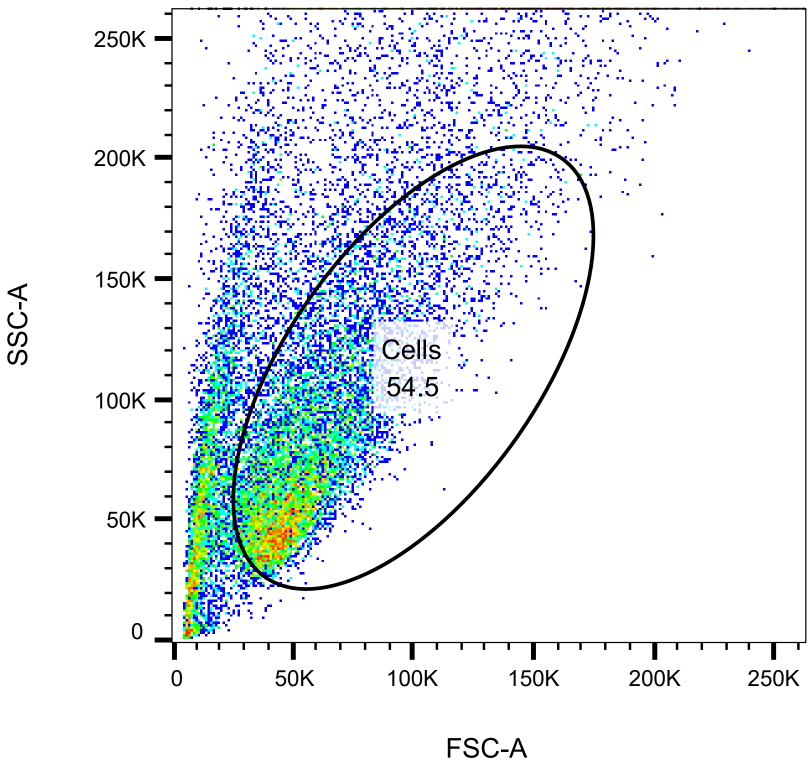

Specimen_001_Naive No UNC Rep1_002.fcs
Ungated
20000

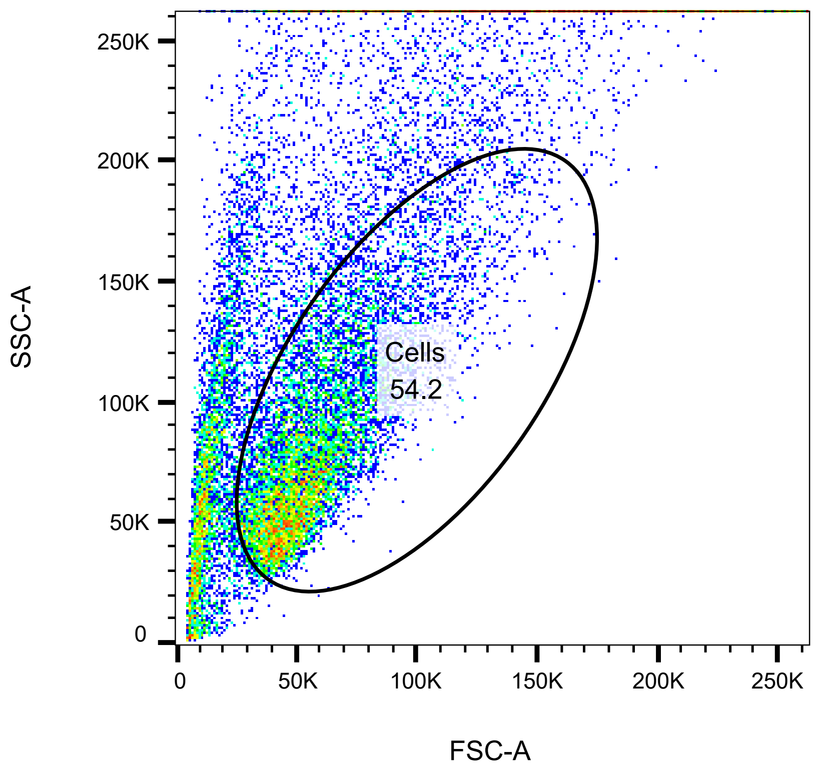

Specimen_001_Naive with UNC Rep1_004.fcs
Ungated
20000

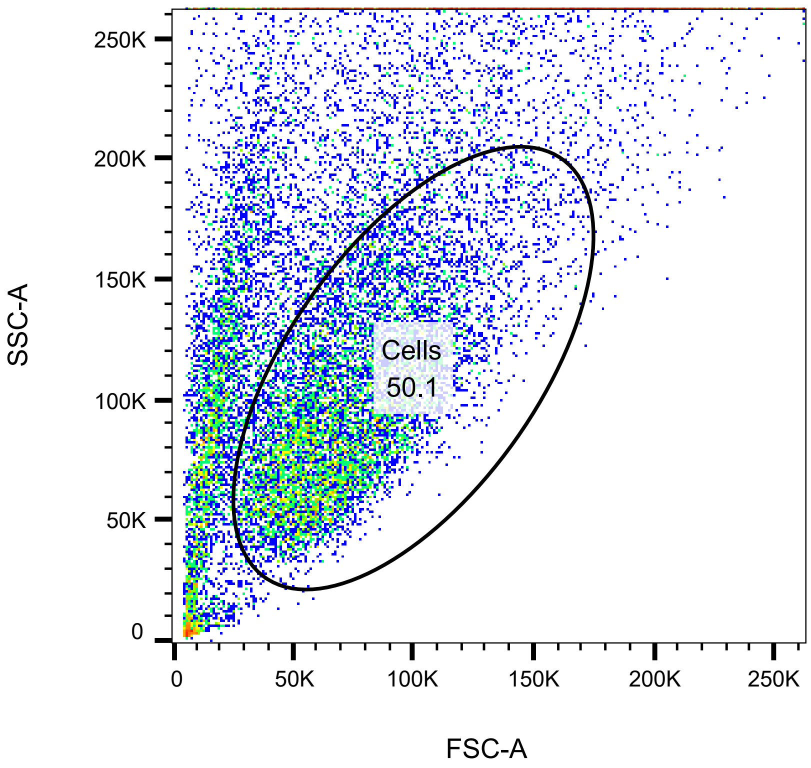

Specimen_001_Naive No UNC Rep2_001.fcs
Ungated
20000

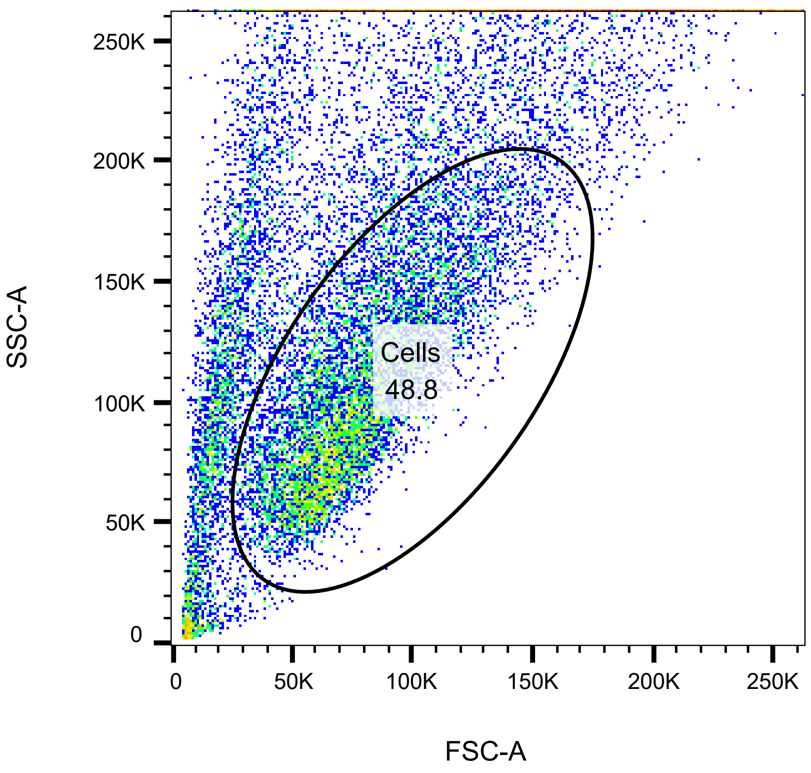

Specimen_001_Naive with UNC Rep2_002.fcs
Ungated
20000

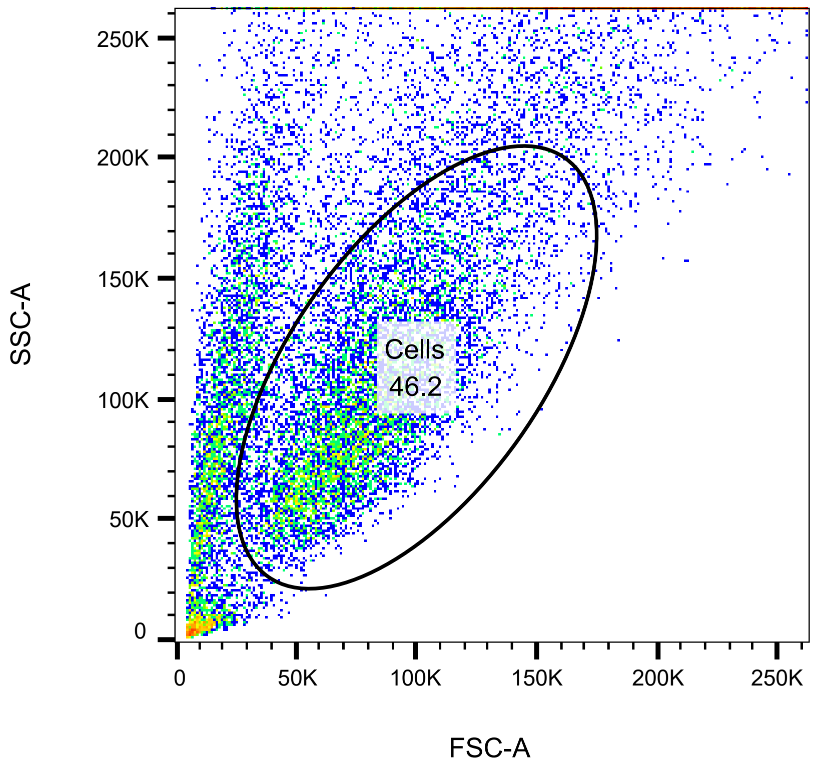

Specimen_001_Naive No UNC Rep3_003.fcs
Ungated
20000

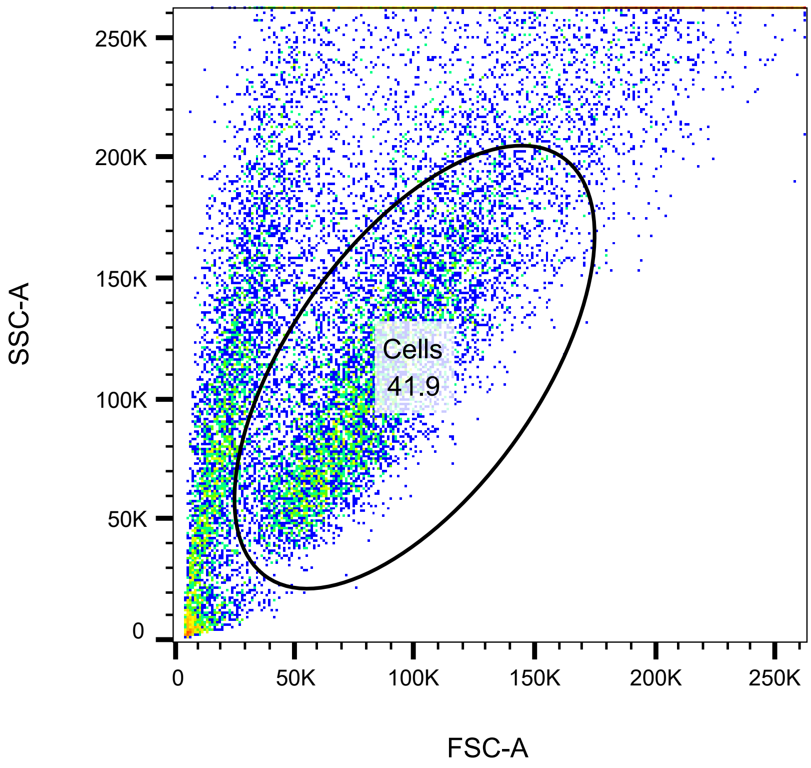

Specimen_001_Naive with UNC Rep3_004.fcs
Ungated
20000

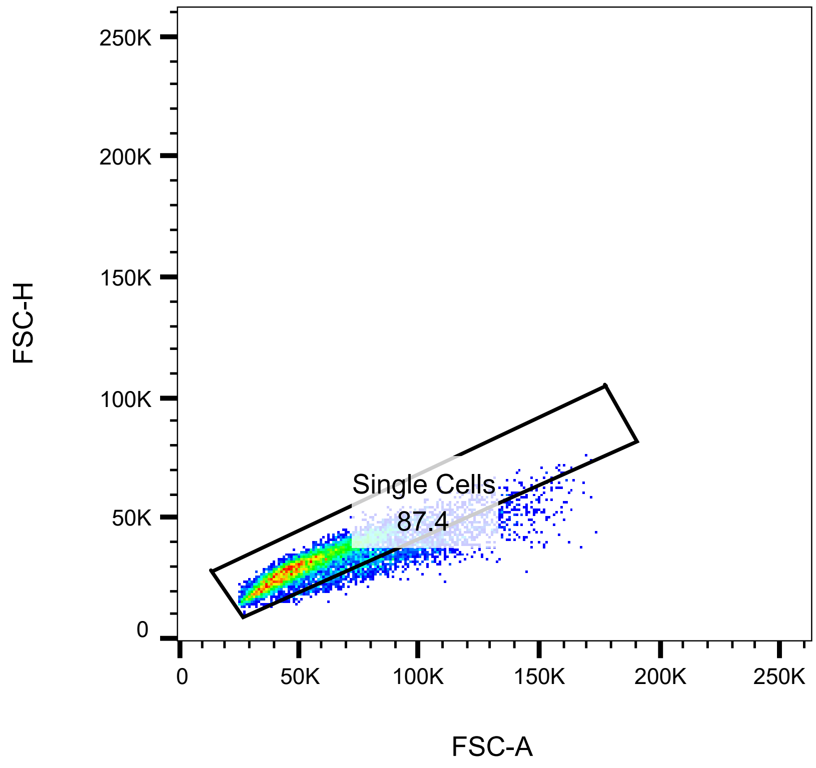

Specimen_001_Naive No UNC Rep1_002.fcs
Cells
10902

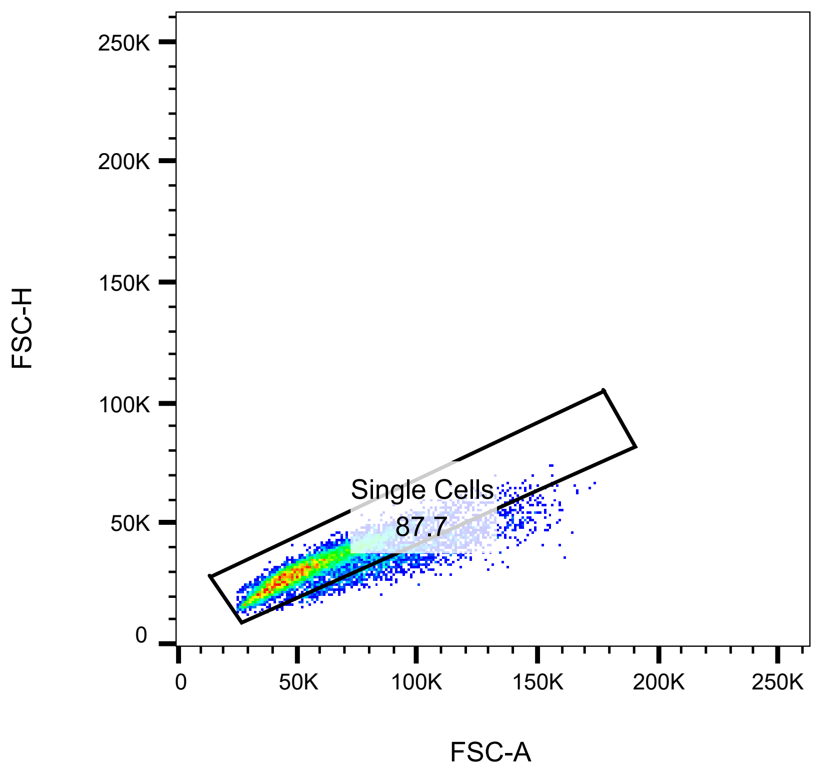

Specimen_001_Naive with UNC Rep1_004.fcs
Cells
10838

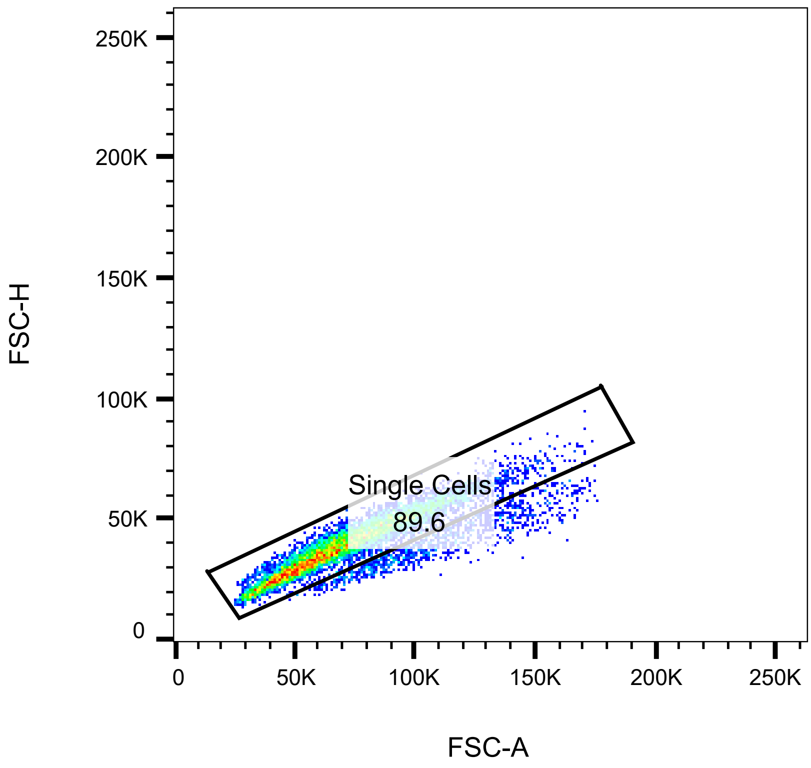

Specimen_001_Naive No UNC Rep2_001.fcs
Cells
10029

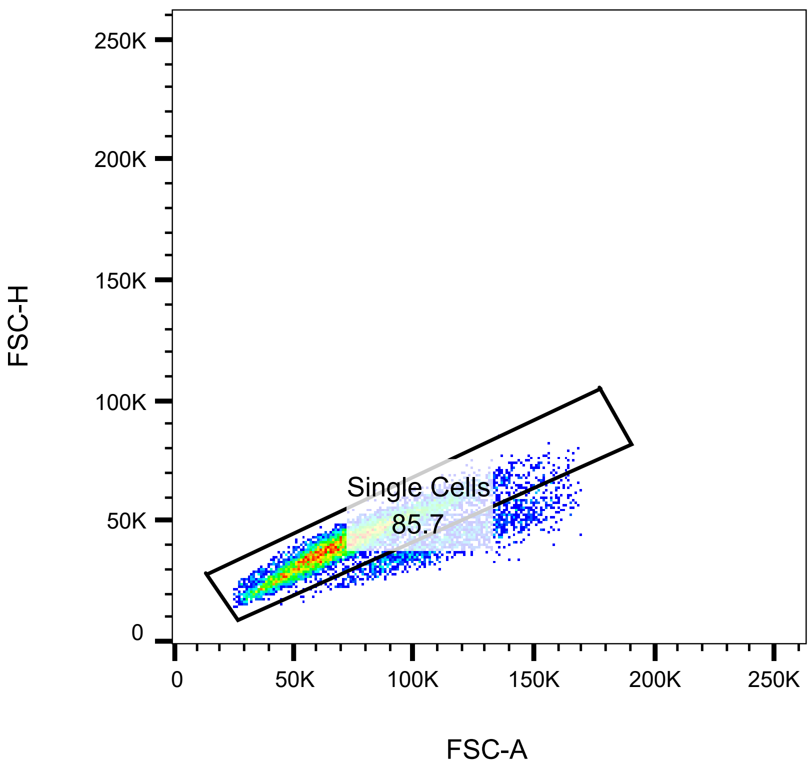

Specimen_001_Naive with UNC Rep2_002.fcs
Cells
9765

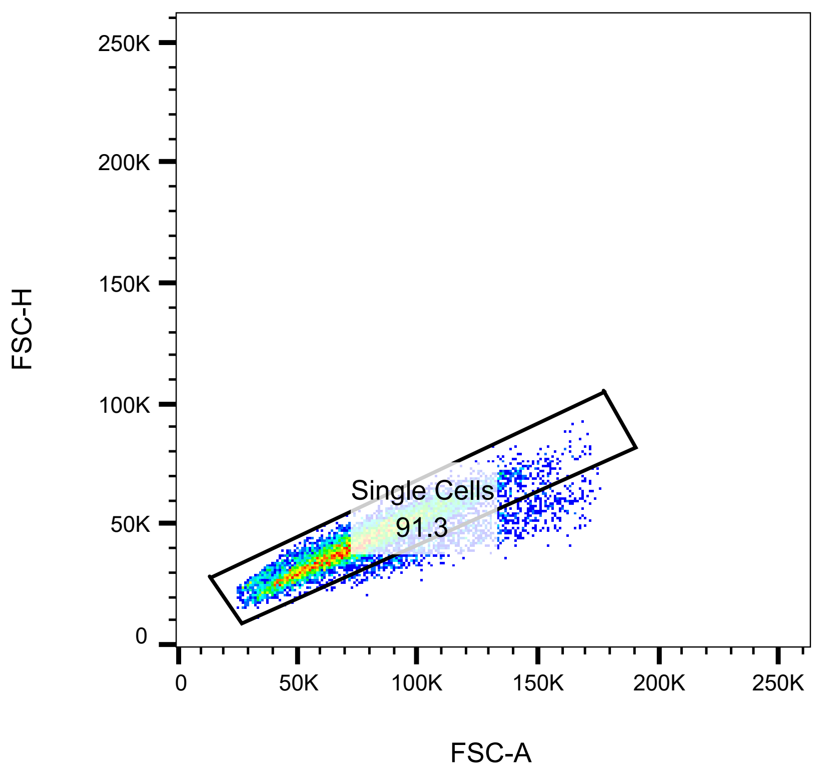

Specimen_001_Naive No UNC Rep3_003.fcs
Cells
9243

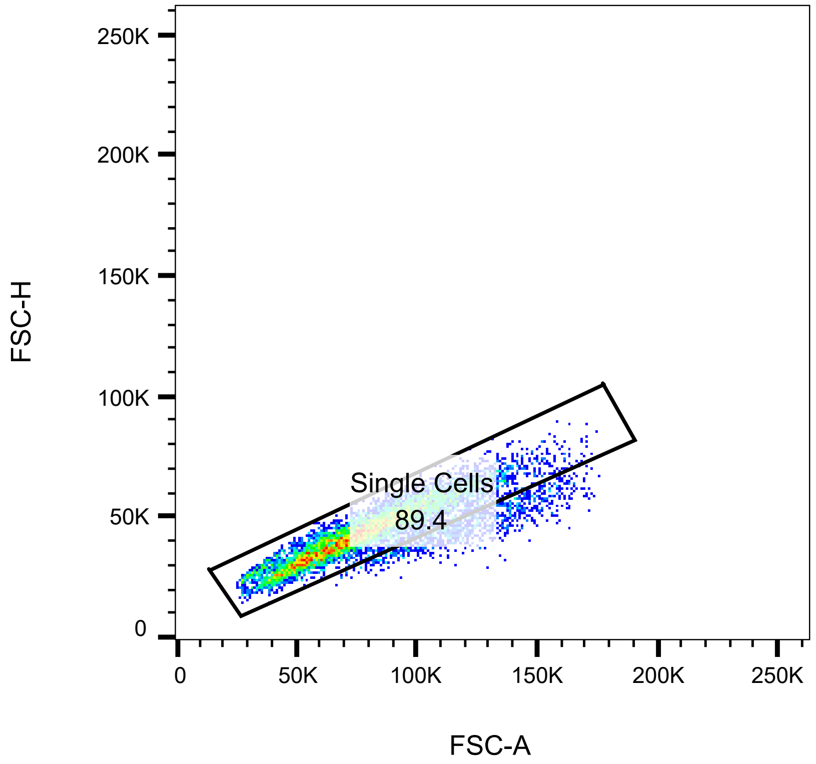

Specimen_001_Naive with UNC Rep3_004.fcs
Cells
8383

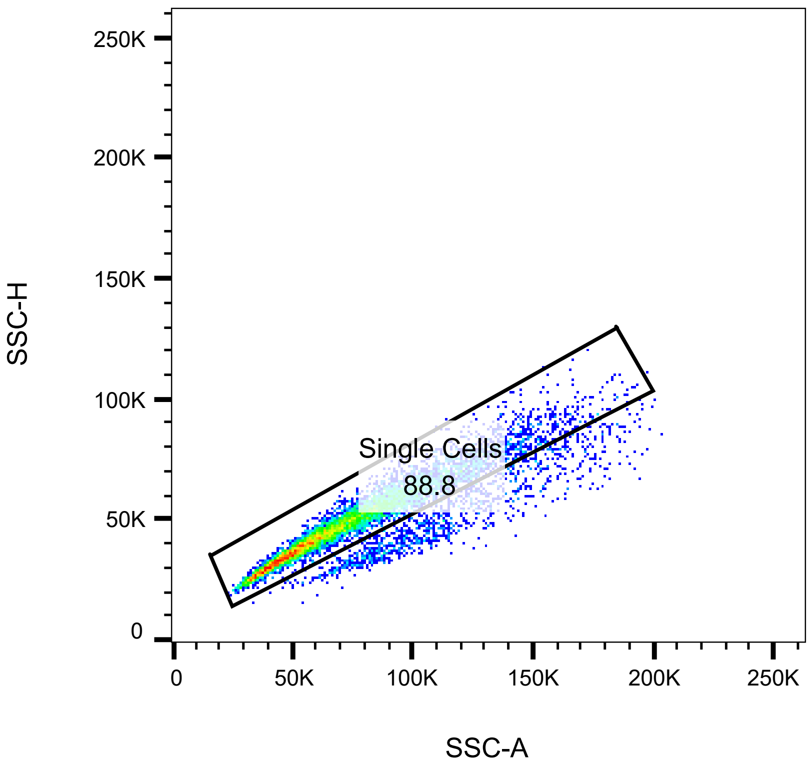

Specimen_001_Naive No UNC Rep1_002.fcs
Single Cells
9533

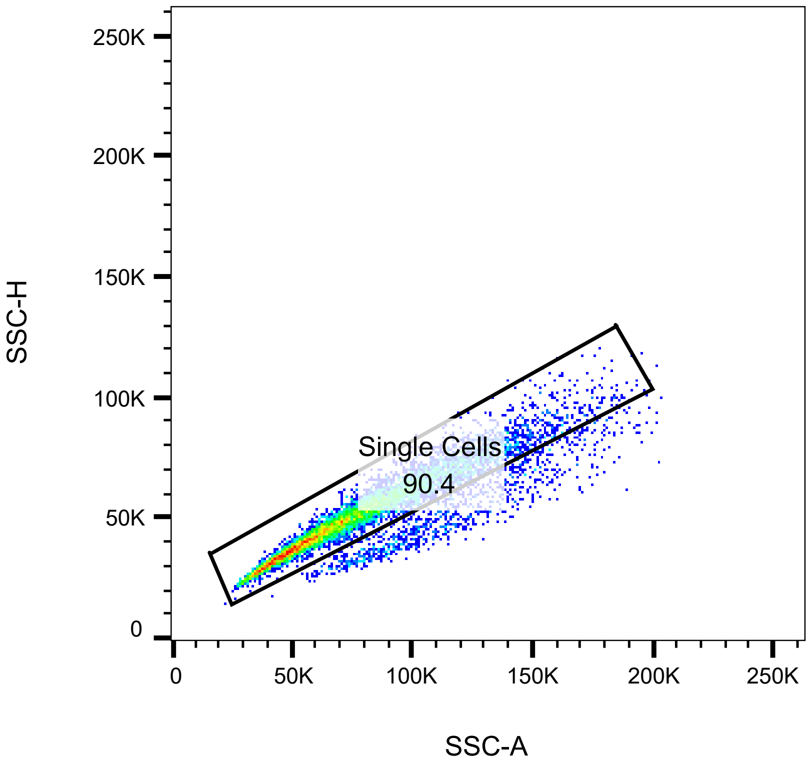

Specimen_001_Naive with UNC Rep1_004.fcs
Single Cells
9502

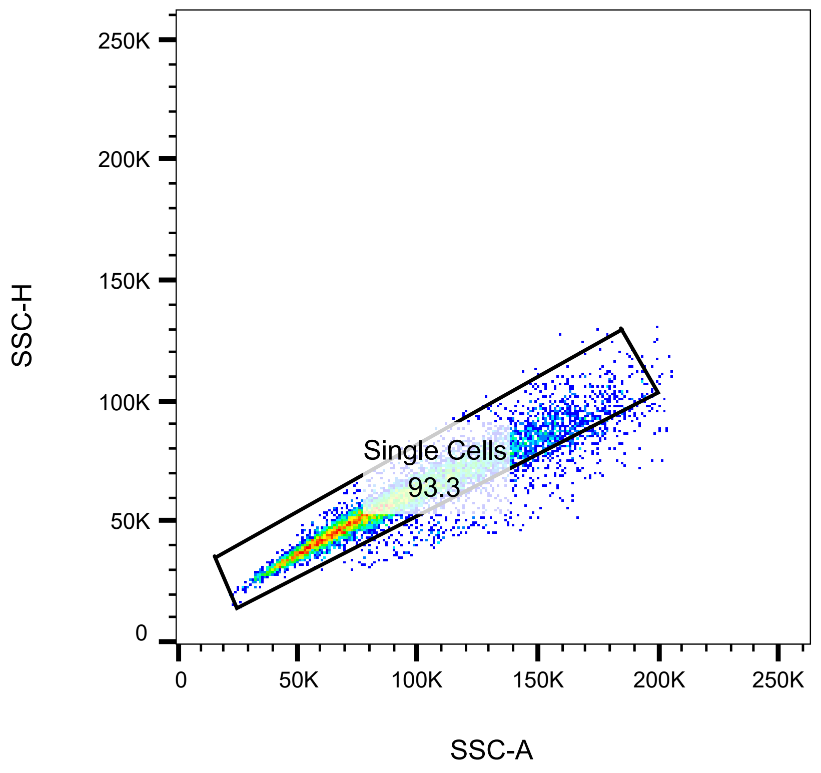

Specimen_001_Naive No UNC Rep2_001.fcs
Single Cells
8990

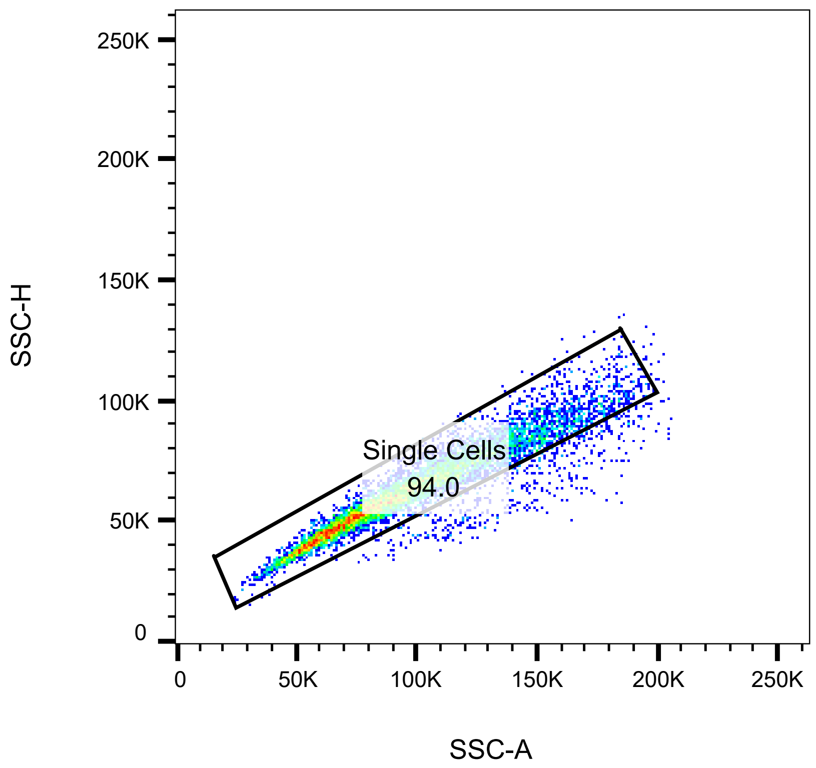

Specimen_001_Naive with UNC Rep2_002.fcs
Single Cells
8369

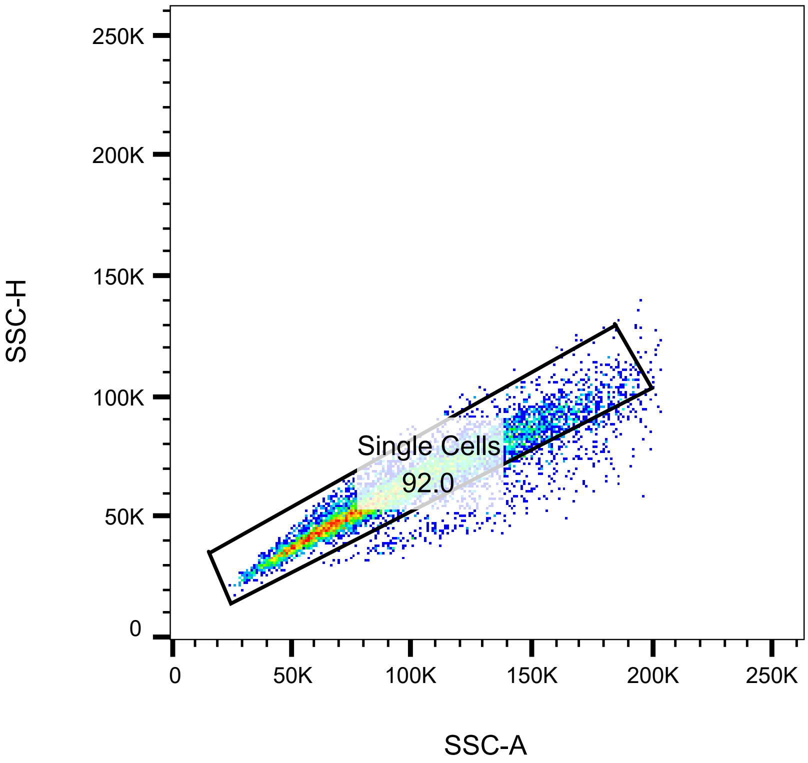

Specimen_001_Naive No UNC Rep3_003.fcs
Single Cells
8435

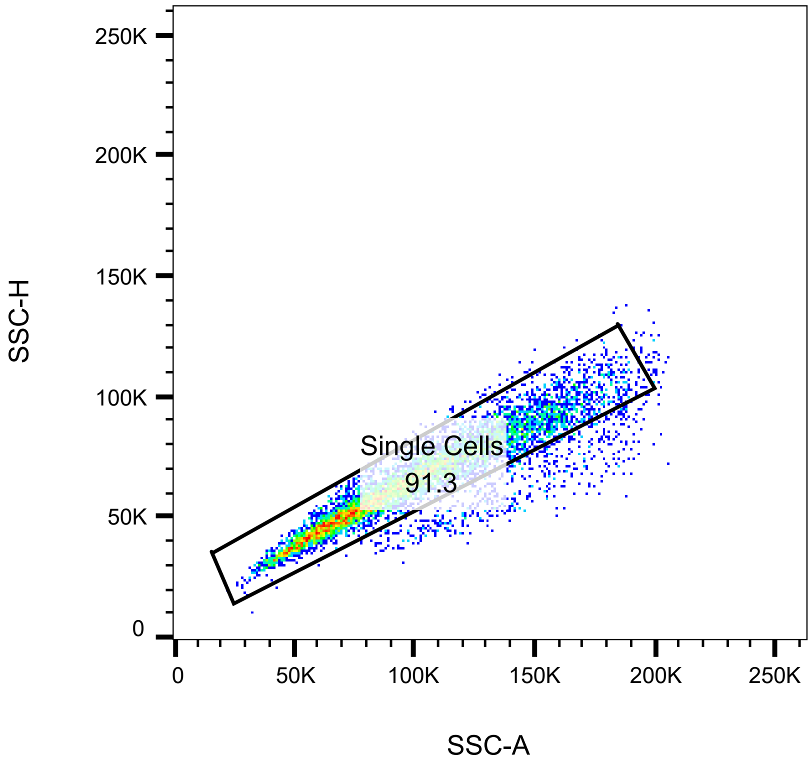

Specimen_001_Naive with UNC Rep3_004.fcs
Single Cells
7498

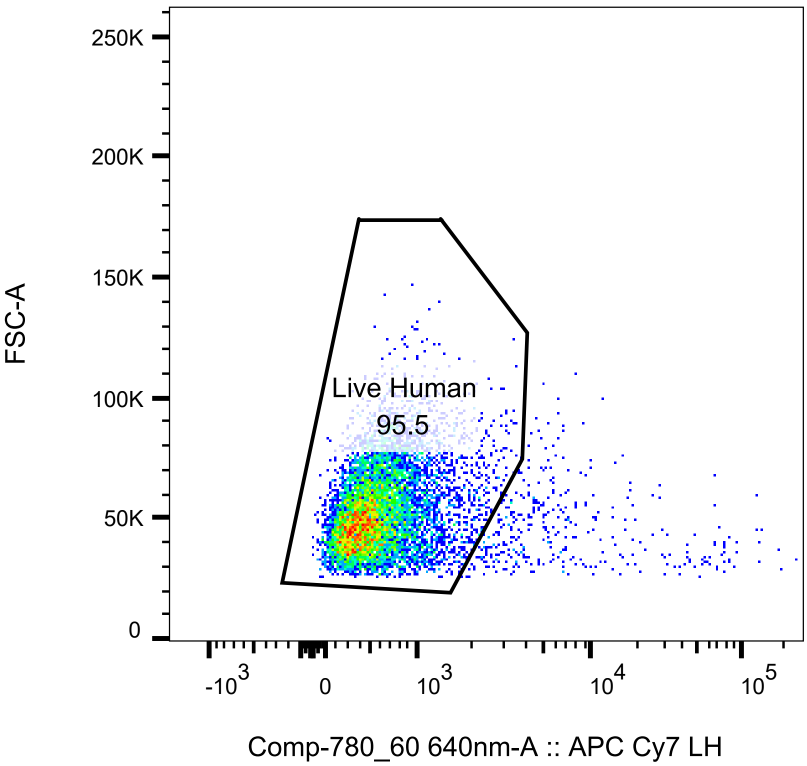

Specimen_001_Naive No UNC Rep1_002.fcs
Single Cells
8464

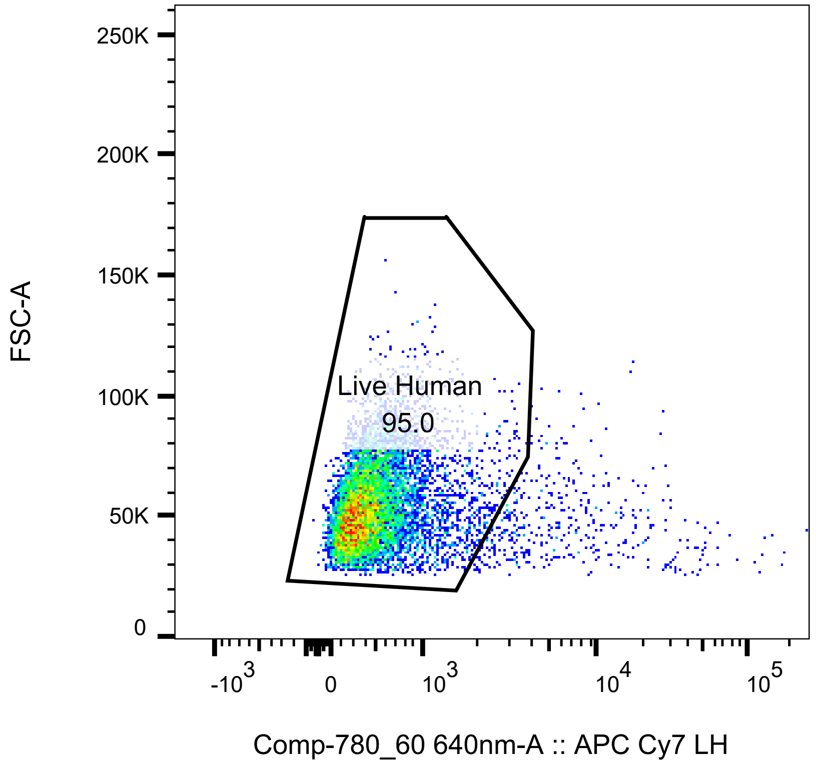

Specimen_001_Naive with UNC Rep1_004.fcs
Single Cells
8588

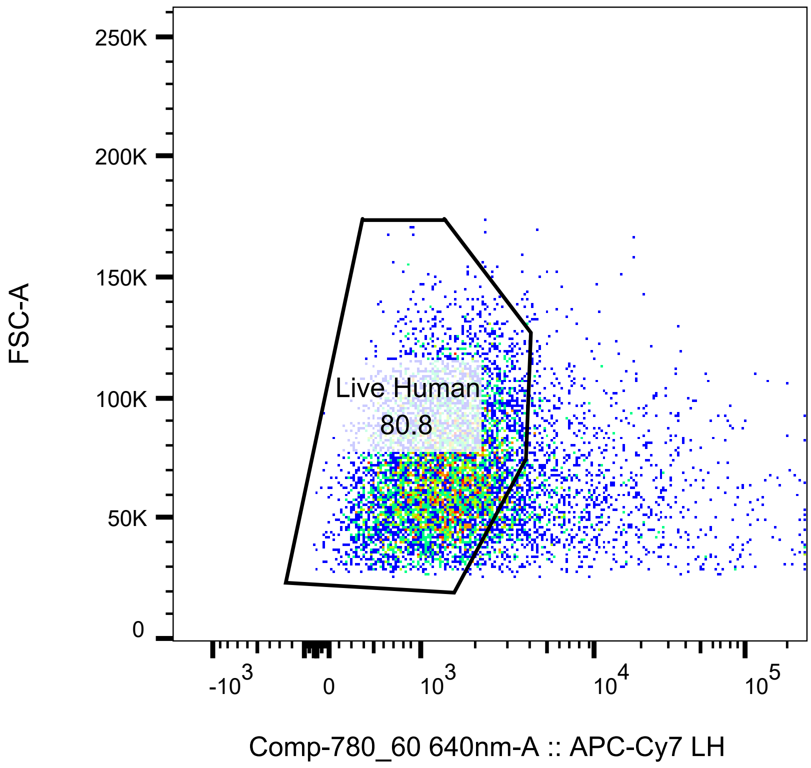

Specimen_001_Naive No UNC Rep2_001.fcs
Single Cells
8391

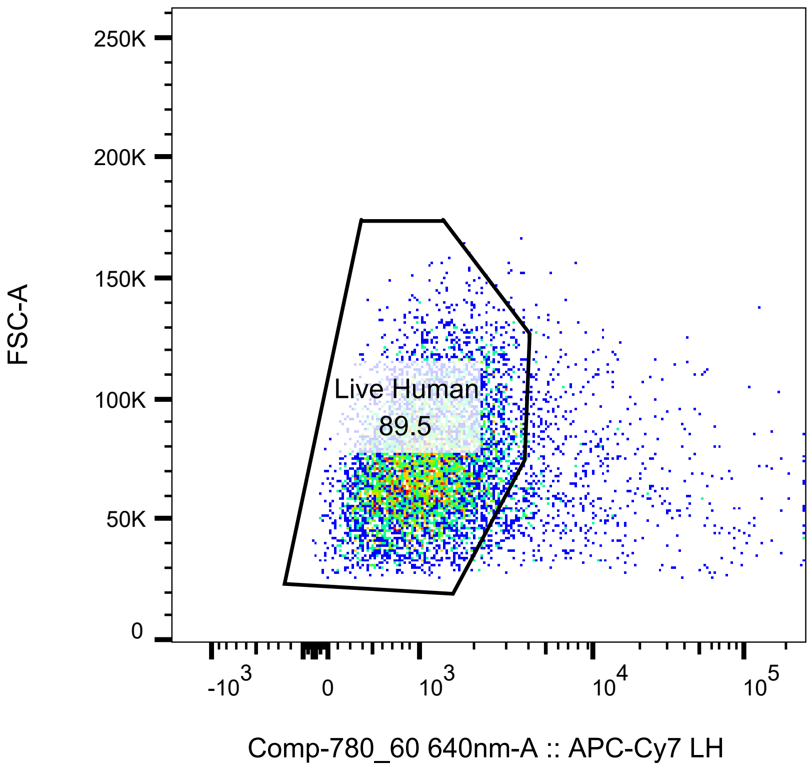

Specimen_001_Naive with UNC Rep2_002.fcs
Single Cells
7871

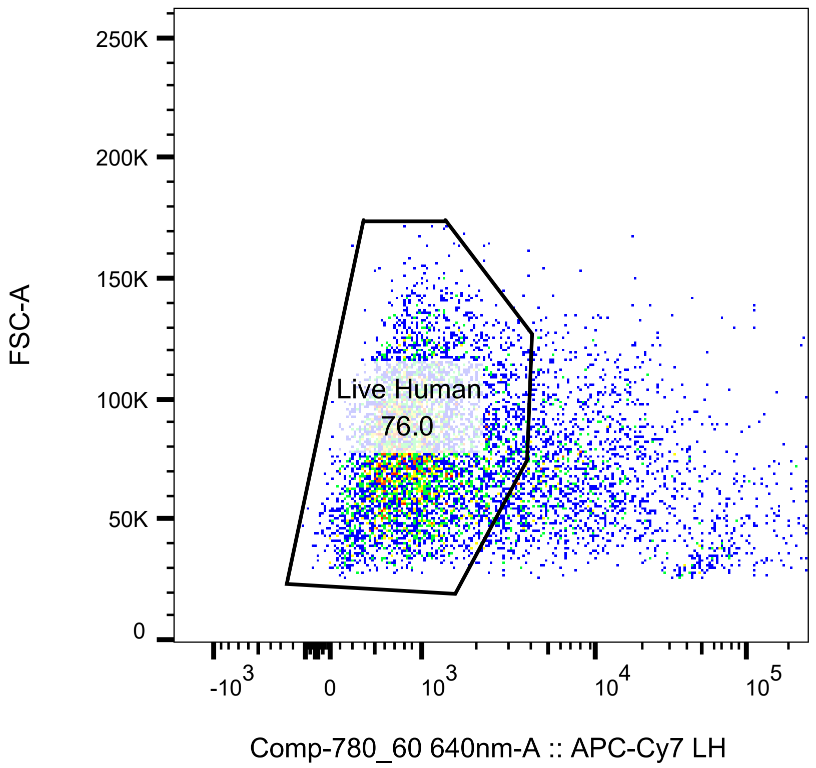

Specimen_001_Naive No UNC Rep3_003.fcs
Single Cells
7764

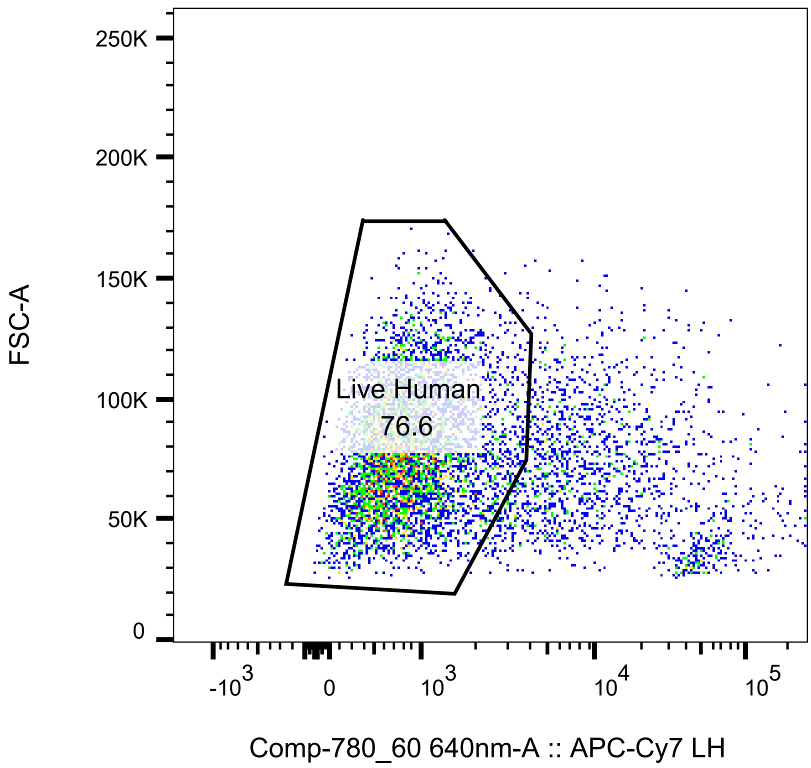

Specimen_001_Naive with UNC Rep3_004.fcs
Single Cells
6842

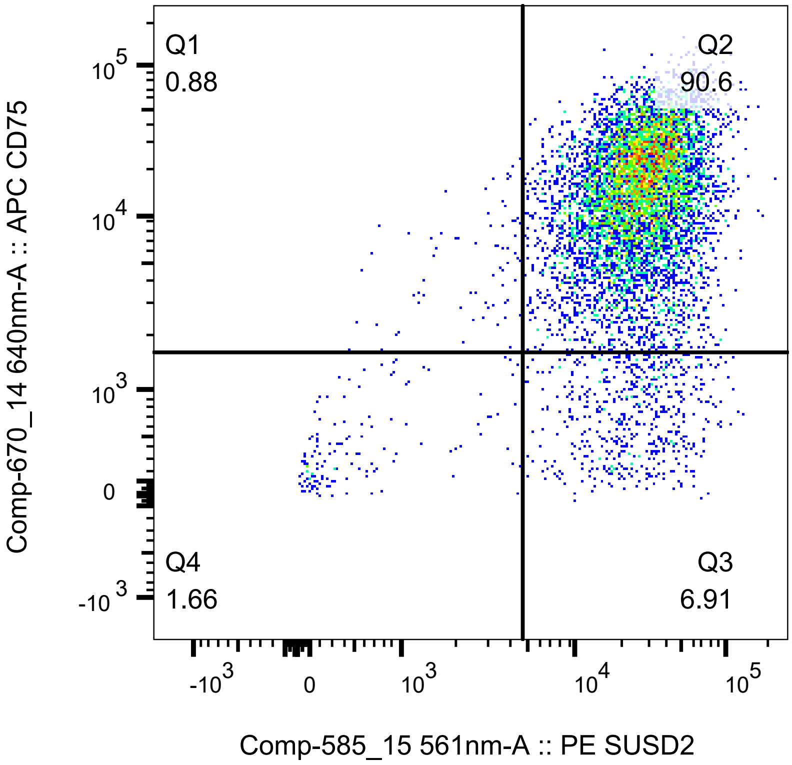

Specimen_001_Naive No UNC Rep1_002.fcs
Live Human
8086

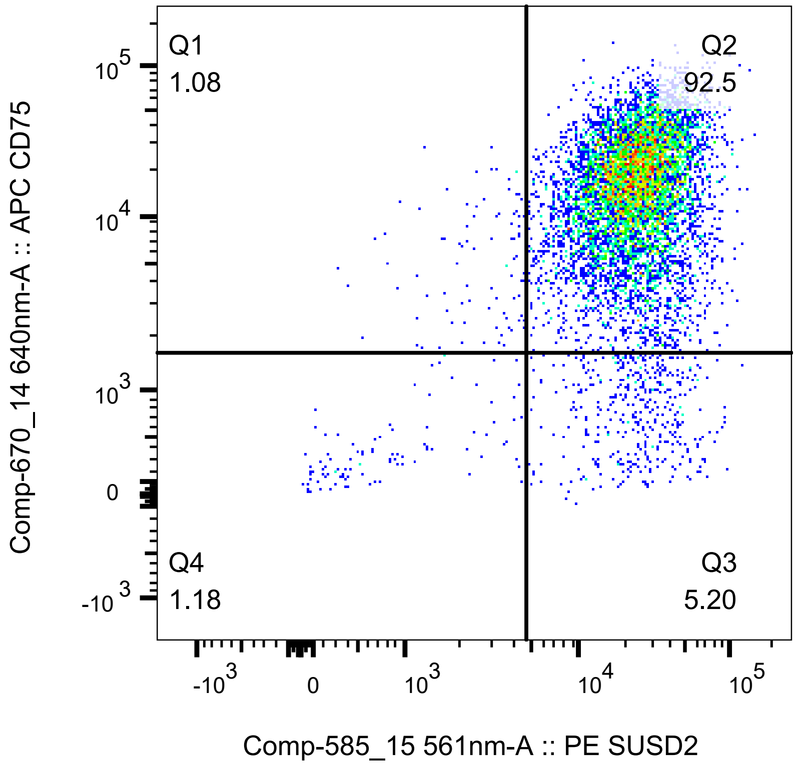

Specimen_001_Naive with UNC Rep1_004.fcs
Live Human
8158

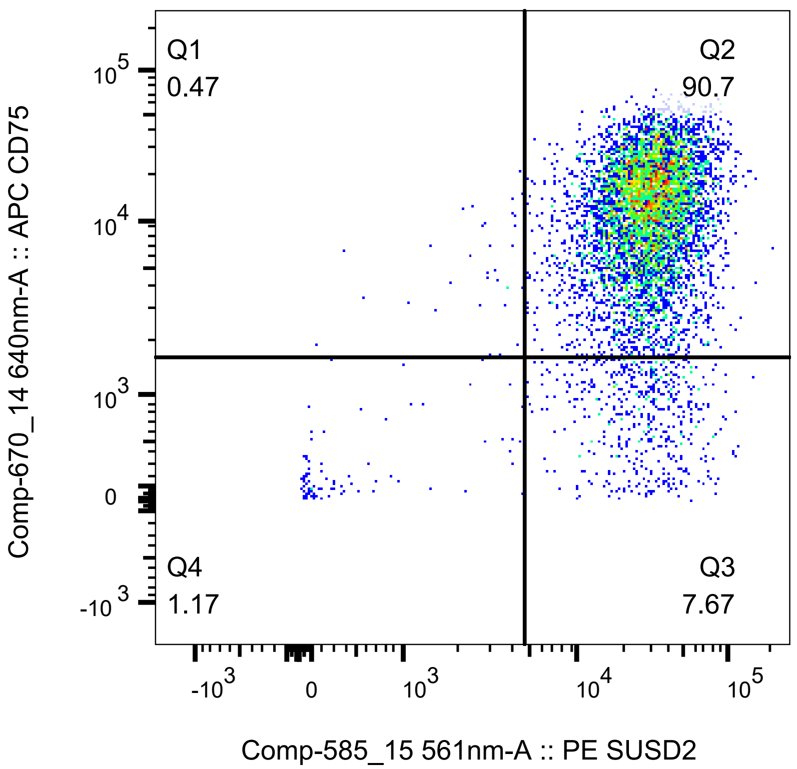

Specimen_001_Naive No UNC Rep2_001.fcs
Live Human
6778

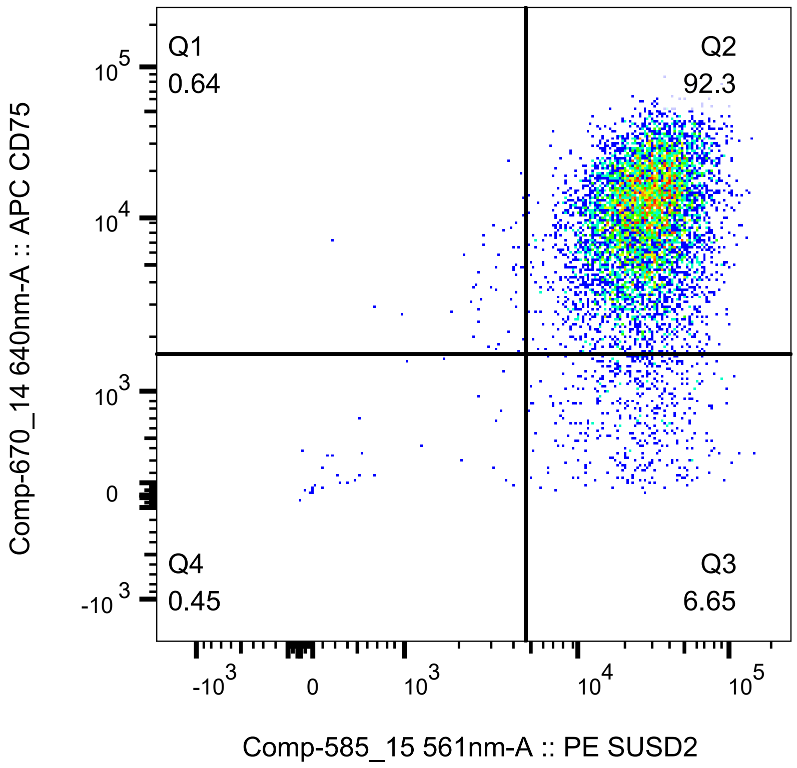

Specimen_001_Naive with UNC Rep2_002.fcs
Live Human
7041

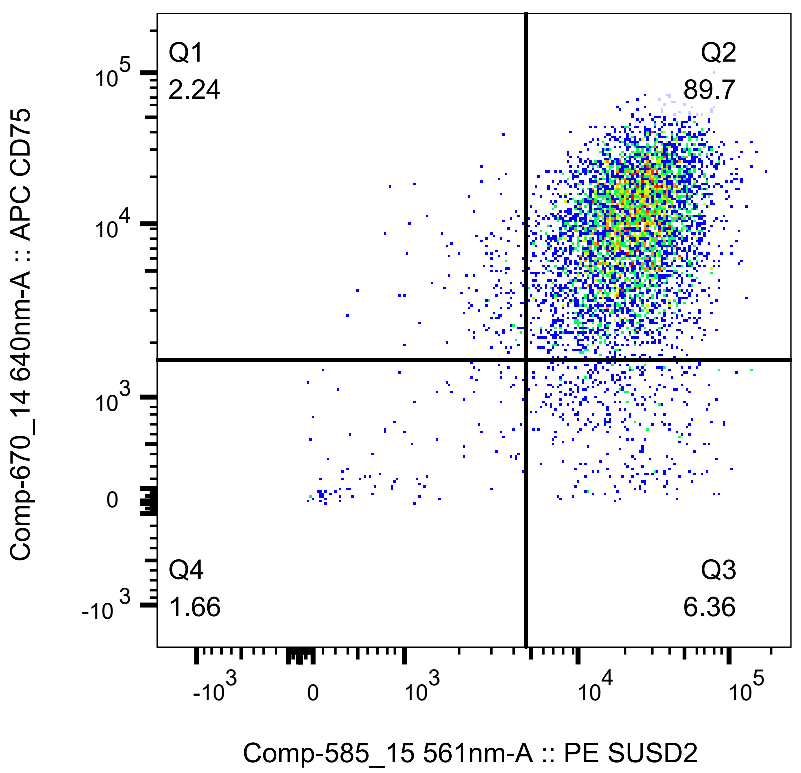

Specimen_001_Naive No UNC Rep3_003.fcs
Live Human
5898

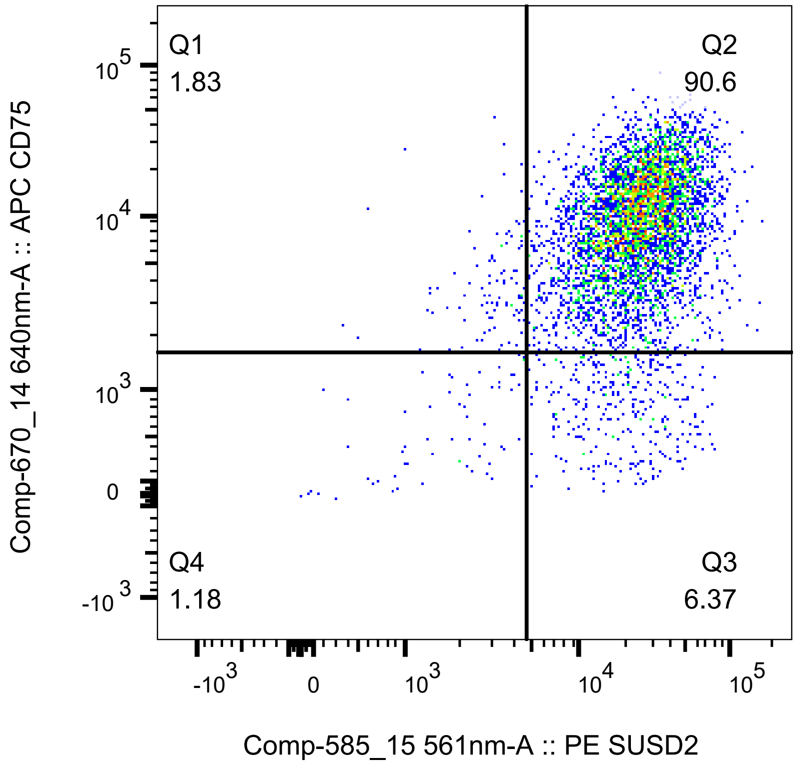

Specimen_001_Naive with UNC Rep3_004.fcs
Live Human
5240

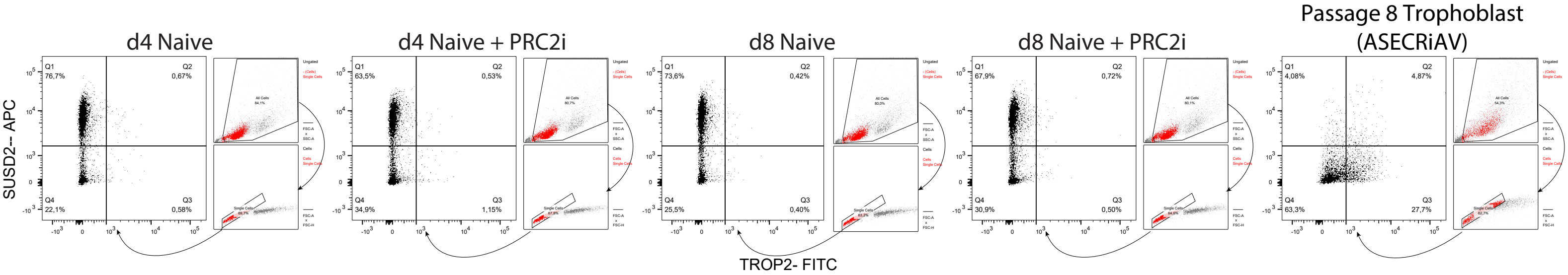

Gating strategy: Forward scatter area (FSC-A) and side scatter area (SSC-A) were used to select for cells from debris (gate labeled all cells, top right). Then FSC-A and forward scatter height (FSC-H) were used to separate single cells from multiplets (gate labeled as single cells, bottom right). The gated single cells were then used in the main plot.

Extended Data Figure 5d

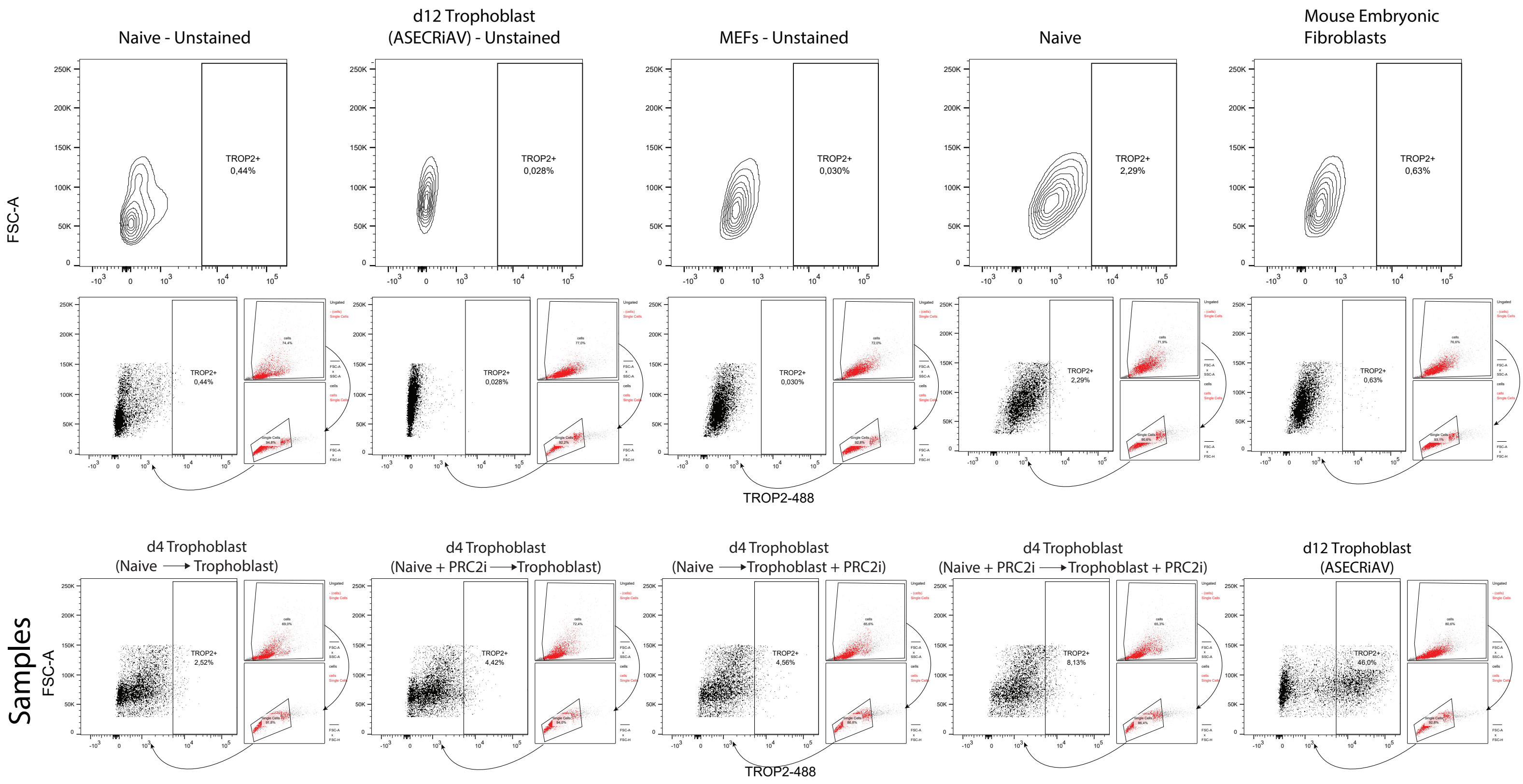

Gating strategy: Forward scatter area (FSC-A) and side scatter area (SSC-A) were used to select for cells from debris (gate labeled all cells, top right). Then FSC-A and forward scatter height (FSC-H) were used to separate single cells from multiplets (gate labeled as single cells, bottom right). The gated single cells were then used in the main plot.

Extended Data Figure 5i

Experiment 1:

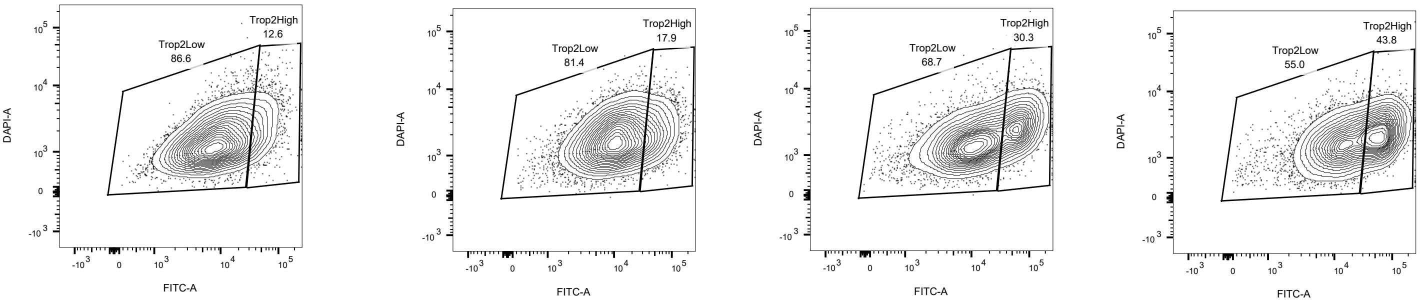

36hrs_blastoid_PRCi_NoPretreatment_withoutPRCi_004.fcs
Single Cells
9769

36hrs_blastoid_PRCi_PretreatedwithPRCi_002.fcs
Single Cells
10013

60hrs_blastoid_PRCi_001_NoPretreatment_withoutPRCi_003.fcs
Single Cells
8106

60hrs_blastoid_PRCi_001_Pretreated_withPRCi_005.fcs
Single Cells
9364

Experiment 2:

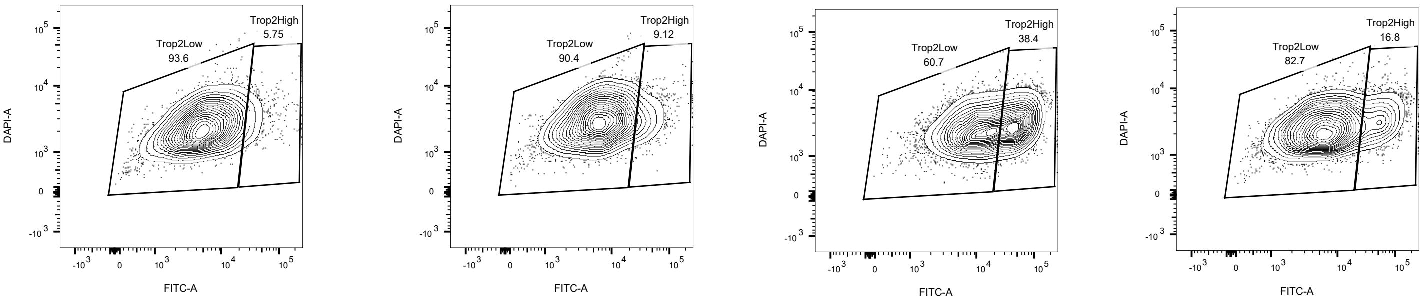

Specimen_001_36hours_Control_002.fcs
Single Cells
5580

Specimen_001_36hours_PRC2i_003.fcs
Single Cells
6263

Specimen_001_60hours_PRC2i_003.fcs
Single Cells
8276

Specimen_001_60hours_Wt_002.fcs
Single Cells
8596

Experiment 3:

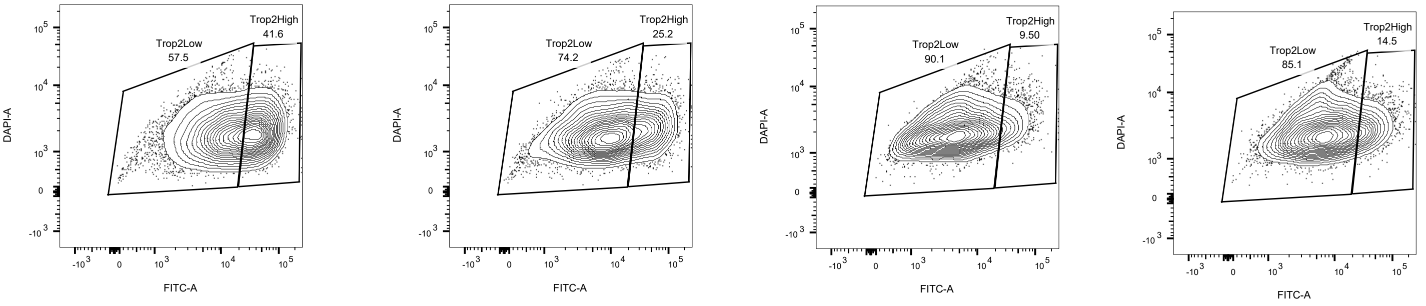

20211211_60hours_prc2i_002.fcs
Single Cells
11206

20211211_60hours_wt_001.fcs
Single Cells
9500

Specimen_001_36hours_wt_002.fcs
Single Cells
9827

Specimen_001_36hours_prc2i_003.fcs
Single Cells
11158

Extended Data Figure 7b
