# Supplementary material for: Integrated multi-omics reveal polycomb repressive complex 2 restricts human trophoblast induction
Source: Nat Cell Biol. 2022 Jun 13;24(6):858–71. doi: 10.1038/s41556-022-00932-w (PMC9203278; doi:10.1038/s41556-022-00932-w)
Supplement: Supplementary file 2 — Reporting Summary [file 41556_2022_932_MOESM2_ESM.pdf]

nature portfolio

Corresponding author(s): Vincent Pasque
Peter J. Rugg-Gunn
Hendrik Marks
Maarten Dhaenens

Last updated by author(s): Apr 4, 2022

Reporting Summary

Nature Portfolio wishes to improve the reproducibility of the work that we publish. This form provides structure for consistency and transparency in reporting. For further information on Nature Portfolio policies, see our Editorial Policies and the Editorial Policy Checklist.

Statistics

For all statistical analyses, confirm that the following items are present in the figure legend, table legend, main text, or Methods section.

n/a	Confirmed	
☐	☒	The exact sample size (n) for each experimental group/condition, given as a discrete number and unit of measurement
☐	☒	A statement on whether measurements were taken from distinct samples or whether the same sample was measured repeatedly
☐	☒	The statistical test(s) used AND whether they are one- or two-sided<br>*Only common tests should be described solely by name; describe more complex techniques in the Methods section.*
☒	☐	A description of all covariates tested
☐	☒	A description of any assumptions or corrections, such as tests of normality and adjustment for multiple comparisons
☐	☒	A full description of the statistical parameters including central tendency (e.g. means) or other basic estimates (e.g. regression coefficient) AND variation (e.g. standard deviation) or associated estimates of uncertainty (e.g. confidence intervals)
☐	☒	For null hypothesis testing, the test statistic (e.g. F, t, r) with confidence intervals, effect sizes, degrees of freedom and P value noted<br>*Give P values as exact values whenever suitable.*
☒	☐	For Bayesian analysis, information on the choice of priors and Markov chain Monte Carlo settings
☒	☐	For hierarchical and complex designs, identification of the appropriate level for tests and full reporting of outcomes
☐	☒	Estimates of effect sizes (e.g. Cohen's d, Pearson's r), indicating how they were calculated

Our web collection on statistics for biologists contains articles on many of the points above.

Software and code

Policy information about availability of computer code

Data collection	CUT&RUN (SEACR) algorithm (v1.3)
Data analysis	FlowJo V10.1 software<br><br>Nikon NIS-Elements AR with DeNoise AI and 3D Richardson-Lucy algorithm (v5.30.01)<br>Axio Vision software (v4.9.1.0)<br>ImageJ (v1.37a)<br><br>Microsoft Excel (Microsoft Office Professional Plus 2016)<br>R (v4.0.3)<br>ggplot2 (v3.3.3)<br>FastQC (v0.11.8; Babraham Bioinformatics)<br>STAR (v2.7.1a)<br>featureCounts (v2.0.1)<br>DESeq2 (v1.26.0)<br>10X Genomics Cell Ranger (v4.0.0)<br>biomaRt (v2.64.3)<br>Seurat (v4.0.1)<br><br>TTrimGalore (v.0.6.6: Babraham Bioinformatics)<br>Bowtie2 (v.2.3.2)<br>DESEQ2 implementation within SeqMonk (v1.47.2; Babraham Bioinformatics)<br>ChIPseeker (v1.30.3)

samtools (v1.11)
bedtools (v2.29.2)
deeptools (v3.43)
WashU epigenome browser (v5)
Seqmonk (v1.47.2: Babraham Bioinformatics)

MaxQuant (v1.6.6.0)
Perseus (v1.5.0.0)
Cytoscape (v3.7.2)

Progenesis QIP 4.2. (Nonlinear Dynamics, Waters)
Mascot Daemon (v2.6.0)
MSqRob (v0.7.6)

Codes pertaining to analyses in this study are available from GitHub webpages:
https://github.com/pasquelab/PRC2 (DOI: 10.5281/zenodo.6398543),
https://github.com/laurabiggins/Shiny_omics (DOI: 10.5281/zenodo.6400749), and
https://github.com/AndrewAMalcolm/Zijlmans-et-al.-2022 (DOI: 10.5281/zenodo.6399297).

For manuscripts utilizing custom algorithms or software that are central to the research but not yet described in published literature, software must be made available to editors and reviewers. We strongly encourage code deposition in a community repository (e.g. GitHub). See the Nature Portfolio guidelines for submitting code & software for further information.

Data

Policy information about availability of data

All manuscripts must include a data availability statement. This statement should provide the following information, where applicable:

- Accession codes, unique identifiers, or web links for publicly available datasets
- A description of any restrictions on data availability
- For clinical datasets or third party data, please ensure that the statement adheres to our policy

Data generated during this study has been deposited in public repositories as follows:

The multi-omic data can be explored using the online resource: https://www.bioinformatics.babraham.ac.uk/shiny/shiny_omics/Shiny_omics
Figures 1, 2, 3, 4 and Extended Data Figures 1, 2, 3, 4.

Raw and processed sequencing data for RNA-seq, cCUT&RUN and scRNA-seq (including scRNA-seq loom files to be visualised on the SCope platform: https://scope.aertslab.org/#/HumanPluripotencyPRC2/*/welcome) have been submitted to the NCBI GEO (http://www.ncbi.nlm.nih.gov/geo/) under accession number GSE176175.
Figures 3, 4, 5, 6 and Extended Data Figures 3, 4, 5, 6.

Raw and processed sequencing data generated in this study (ChEP mass spectrometry proteomics data):
PRIDE partner repository, https://www.ebi.ac.uk/pride/
(dataset identifier PXD028111)
Figures 1, 2, 4 and Extended Data Figures 1, 2, 4.

Raw and processed sequencing data generated in this study (hPTM mass spectrometry proteomics):
PRIDE partner repository, https://www.ebi.ac.uk/pride/
(dataset identifier PXD028162, 10.6019/PXD028162 and PXD032792)
Figures 2, 4 and Extended Data Figures 2, 4.

Public databases/datasets used in this manuscript:
Human Swissprot database (https://www.uniprot.org/)
cRAP database (https: //www.thegpm.org/crap/)
HIstome database (http://www3.iiserpune.ac.in/~coee/histome/)
UniProtKB human proteome (https://www.uniprot.org/)
ArrayExpress under the accession number E-MTAB-39293 (Petropoulos et al., 2016)
GEO database under accession number GSE10955589 (Zhou et al., 2019)
van Mierlo et al., 2019; PRIDE partner repository, https://www.ebi.ac.uk/pride/ (dataset identifier PXD007154).

DATA AVAILABILITY
The multi-omic data can be explored using the online resource: https://www.bioinformatics.babraham.ac.uk/shiny/shiny_omics/Shiny_omics. The scRNA-seq loom files can be visualised on the SCope platform: https://scope.aertslab.org/#/HumanPluripotencyPRC2/*/welcome.
RNA sequencing, cCUT&RUN, and single-cell RNA sequencing datasets have been deposited in the Gene Expression Omnibus (GEO) under the accession code of GSE176175.
hPTM mass spectrometry proteomics datasets have been deposited in the ProteomeXchange Consortium via the PRIDE partner repository (https://www.ebi.ac.uk/pride/) under the dataset identifiers PXD028162, 10.6019/PXD028162 and PXD032792. The project with identifier PXD028162 (consultable via ProteomeXchange) was licensed on a single run basis and is fully accessible and editable by the readership after free download of the Progenesis QIP 4.2 software (https://www.nonlinear.com/progenesis/qi-for-proteomics/). ChEP mass spectrometry proteomics datasets have been deposited in the ProteomeXchange Consortium via the PRIDE partner repository (https://www.ebi.ac.uk/pride/) under the dataset identifier PXD028111. Furthermore, datasets were downloaded as provided by Petropoulos et al. (ArrayExpress: E-MTAB-39293), Zhou et al. (Zhou et al., 2019) (GEO: GSE10955580) and van Mierlo et al. (GEO: GSE101675). Public databases used in this manuscript include Human Swissprot database (https://www.uniprot.org/), cRAP database (https: //www.thegpm.org/crap/), HIstome database (http://www3.iiserpune.ac.in/~coee/histome/), UniProtKB human proteome (https://www.uniprot.org/). Source data are provided with this paper. All other data supporting the findings of this study are available from the corresponding authors on reasonable request.

Field-specific reporting

Please select the one below that is the best fit for your research. If you are not sure, read the appropriate sections before making your selection.

☒ Life sciences ☐ Behavioural & social sciences ☐ Ecological, evolutionary & environmental sciences

For a reference copy of the document with all sections, see nature.com/documents/nr-reporting-summary-flat.pdf

Life sciences study design

All studies must disclose on these points even when the disclosure is negative.

Sample size	All sample size are indicated on the figure legends or in the manuscript.<br>In stem cells, qPCR, flow cytometry, western blotting and immunostaining, experiments were performed in two or three independent replicates unless stated in figure legends.<br>In blastoids experiments, flow cytometry and cavity formation experiments were performed in three independent experiments.<br>Immunofluorescence in blastoids were performed in several blastoids from one independent experiment.<br>In bulk RNAseq, ChEP, and cCUT&RUN experiments were performed in two or three biological replicates.<br>hPTM mass spectrometry was performed for at least five biological replicates per condition.<br>No statistical method was used to determine sample size. Sample sizes were chosen based on previous experience from similar studies.
Data exclusions	For ChEP, proteins flagged as 'reverse', 'potential contaminant' or 'only identified by site' were filtered from the final protein list. Biological triplicates were grouped to calculate differential proteins. Data were filtered for 3 valid values in at least 1 group. Missing values were imputed using default settings in Perseus, based on the assumption that they were not detected because they were under or close to detection limit.<br>For bulk RNAseq analysis samples were filtered to keep genes that had more than 1 count in at least 2 conditions.<br>For single-cell RNAseq analysis human cells were retained and mouse cells (MEFs) were filtered out by adjusting the number of counts per cell (nCount_RNA) and the number of mapped genes per cell (nFeature_RNA) to only keep cells that were mostly mapped to the human GRCh38 (hg38) genome (for naive cells: nCount_RNA < 40000, nCount_RNA > 3000, nFeature_RNA < 8000 and nFeature_RNA > 1500; for day 4 of naive to trophoblast conversions: nCount_RNA < 300000, nCount_RNA > 10000, nFeature_RNA < 12000 and nFeature_RNA > 3000). Naive cells with more than 25% of mitochondrial counts were filtered out. Day 4 trophoblast converted cells with more than 30% of mitochondrial counts were filtered out.<br>For the hPTM analysis, outliers were removed first on the basis of the normalisation factor in Progenesis QIP 4.2: a normalisation factor less than 0,5 or more than 2 were filtered out. Secondly, outliers were removed based on the principal component analysis (PCA).<br>For calibrated CUT&RUN analysis, low quality reads with a MAPQ value > 20 were removed by filtering with samtools view.<br>All exclusion criteria used in this manuscript were pre-established.
Replication	All of the main experiments listed on this manuscript have been successfully replicated at least twice as independent experiments. When this is not the case, this has been indicated in figure legends.
Randomization	For scRNA-seq we used canonical correspondance analysis to remove potential technical/sample-to-sample effects and variation.<br>For the mass spectrometry experiments, all samples were run in a randomized fashion. Sample allocation was random in all other experiments
Blinding	ChEP, Acid Extractome, RNA-seq, Western blot, scRNA-seq, imaging<br>Samples from naive versus prime hPSCs, untreated or treated with UNC1999 inhibitor, were used, hence the analyses could not be performed with blinding. No blinding was performed because none of the analyses reported involved procedures that could be influenced by investigator bias.<br><br>Imaging.<br>Quantification of immunostaining experiments were performed by different researchers to confirm scoring.<br><br>For blastoids lineage analysis, counting of nuclei was performed blindly using automated nuclei scoring based on fluorescence using NIS-Elements AR 5.30.01 via a GA3 script.

Reporting for specific materials, systems and methods

We require information from authors about some types of materials, experimental systems and methods used in many studies. Here, indicate whether each material, system or method listed is relevant to your study. If you are not sure if a list item applies to your research, read the appropriate section before selecting a response.

Materials & experimental systems

n/a	Involved in the study
☐	☒ Antibodies
☐	☒ Eukaryotic cell lines
☒	☐ Palaeontology and archaeology
☐	☒ Animals and other organisms
☒	☐ Human research participants
☒	☐ Clinical data
☒	☐ Dual use research of concern

Methods

n/a	Involved in the study
☐	☒ ChIP-seq
☐	☒ Flow cytometry
☒	☐ MRI-based neuroimaging

Antibodies

Antibodies used

Immunostaining:
Anti-NANOG Mouse, 1 in 50, BD Pharmingen, 560482 (clone N31-355); Anti-mouse-Alexa Fluor 488; 555; 647 Donkey, 1 in 500, Invitrogen, A21202; A31570; A31571.
Anti-KLF17 Rabbit, 1 in 333, Abcam, HPA0246329; Anti-rabbit-Alexa Fluor 647 Donkey, 1 in 500, Invitrogen, A31573
Anti-GATA3 Rabbit, 1 in 250, Abcam, ab199428 [clone EPR16651]; Anti-rabbit-Alexa Fluor 647 Donkey, 1 in 500, Invitrogen, A31573
Anti-GATA3 Rat, 1 in 100, Thermo Fisher, 14-9966-82 (clone TWAJ); Anti-rat-Alexa Fluor 488 Donkey, 1 in 500, Invitrogen, A21208
Anti-AQP3 Rabbit, 1 in 200, Antibodies-online.com, ABIN863208; Anti-rabbit-Alexa Fluor 488 Donkey, 1 in 500, Invitrogen, A21206
Anti-FOXA2 Goat, 1 in 40, r&D Systems biotechne, AF2400; Anti-goat-Alexa 546 Donkey, 1 in 500, Invitrogen, A11056
Ant-GATA4 Rat, 1 in 400, eBioscience, 14-9980-82 (clone eBioEvan); Anti-rat-Alexa Fluor 488 Donkey, 1 in 500, Invitrogen, A21208

Flow Cytometry:
Anti-TROP2 Mouse, 1 in 100, R&D Systems, MAB650 (clone 77220); Anti-mouse-Alexa Fluor 488 Donkey, 1 in 500, Invitrogen, A21202
Anti-TROP2-488 Mouse, 1 in 25, R&D systems, FAB650G (clone 77220)
Anti-SUSD2-APC Mouse, 1 in 50, Miltenyi Biotec, 130-121-134 (clone W5C5)
Anti-SUSD2-PE Mouse, 1 in 100, Miltenyi Biotec, 130-111-641 (clone REA795)
Anti-CD75-eF660 Mouse, 1 in 20, eBioscience, 15519896 (clone LN-1)
Anti-SSEA4-APC Mouse, 1 in 40, R&D Systems, FAB1435A (clone MC-813-70)
Anti-CD24-BUV395 Mouse, 1 in 40, BD Bioscience, 563818 (clone ML5)

Calibrated CUT&RUN:
Tri-Methyl-Histone H3 Lysine 27 Rabbit, 1 in 50, Cell Signaling Technology, 9733 (clone C36B11)
Anti-IgG Rabbit, 1 in 50, Invitrogen, 31188
Anti-Histone H2Av spike-in antibody, 1 in 50, Active Motif, 61686
Western Blotting:
Anti-Tri-Methyl-Histone H3 Lysine 27 Rabbit, 1 in 1000, Cell Signaling Technology, 9733 (clone C36B11); Anti-Rabbit IgG (H+L)-DyLight-800 Donkey, 1 in 10000, Invitrogen, SA5-10044
Anti-Rabbit IgG (H+L)-HRP Goat, 1 in 10000, Bio-Rad, 1706515
Western Blotting Anti-Histone H2B Mouse, 1 in 1000, Abcam, ab64165 (clone mAbcam 64165); Anti-Mouse IgG (H+L)-DyLight-680 Donkey, 1 in 10000, Invitrogen, SA5-10170
Anti-Mouse IgG (H+L)-HRP Goat, 1 in 10000, Bio-Rad, 1706516

Validation

Immunostaining:
Anti-NANOG Mouse, 1 in 400, BD Pharmingen, 560482 (clone N31-355)
Anti-mouse-Alexa Fluor 488; 555; 647 Donkey, 1 in 500, Invitrogen, A21202; A31570; A31571
Anti-KLF17 Rabbit, 1 in 200-1 in 500, Abcam, HPA0246329
Anti-rabbit-Alexa Fluor 647 Donkey, 1 in 200-1 in 2000, Invitrogen, A31573
Anti-GATA3 Rabbit, 1 in 250, Abcam, ab199428 [clone EPR16651]
Anti-GATA3 Rat, 1 in 100, Thermo Fisher, 14-9966-82 (clone TWAJ)
Anti-rat-Alexa Fluor 488 Donkey, 1 in 2000, Invitrogen, A21208
Anti-AQP3 Rabbit, 1 in 200, Antibodies-online.com, ABIN863208
Anti-rabbit-Alexa Fluor 488 Donkey, 1 in 1000, Invitrogen, A21206
Anti-FOXA2 Goat, 1 in 13,3-1 in 40, r&D Systems biotechne, AF2400
Anti-goat-Alexa 546 Donkey, 1 in 200-1 in 2000, Invitrogen, A11056
Ant-GATA4 Rat, 1 in 200-1 in 400, eBioscience, 14-9980-82 (clone eBioEvan)

Flow Cytometry:
Anti-TROP2 Mouse, 0.25 µg/10^6 cells, R&D Systems, MAB650 (clone 77220)
Anti-mouse-Alexa Fluor 488 Donkey, no validation for Flow Cytometry, Invitrogen, A21202
Anti-TROP2-488 Mouse, 10 µL/10^6 cells, R&D systems, FAB650G (clone 77220)
Anti-SUSD2-APC Mouse, 1 in 50, Miltenyi Biotec, 130-121-134 (clone W5C5)
Anti-SUSD2-PE Mouse, 1 in 50, Miltenyi Biotec, 130-111-641 (clone REA795)
Anti-CD75-eF660 Mouse, 5µl/Test, eBioscience, 15519896 (clone LN-1)
Anti-SSEA4-APC Mouse, 10 µL/10^6 cells, R&D Systems, FAB1435A (clone MC-813-70)
Anti-CD24-BUV395 Mouse, 5µl/Test, BD Bioscience, 563818 (clone ML5)

Calibrated CUT&RUN:
Tri-Methyl-Histone H3 Lysine 27 Rabbit, 1 in 50, Cell Signaling Technology, 9733 (clone C36B11)

Anti-IgG Rabbit, no validation for CUT&RUN, Invitrogen, 31188
Anti-Histone H2Av spike-in antibody, 1 in 50, Active Motif, 61686
Western Blotting:
Anti-Tri-Methyl-Histone H3 Lysine 27 Rabbit, 1 in 1000, Cell Signaling Technology, 9733 (clone C36B11)
Anti-Rabbit IgG (H+L)-DyLight-800 Donkey, 1 in 5000-1 in 20000, Invitrogen, SA5-10044
Anti-Rabbit IgG (H+L)-HRP Goat, 1 in 3000, Bio-Rad, 1706515
Western Blotting Anti-Histone H2B Mouse, 1 in 200-1 in 1000, Abcam, ab64165 (clone mAbcam 64165)
Anti-Mouse IgG (H+L)-DyLight-680 Donkey, 1 in 1000-1 in 20000, Invitrogen, SA5-10170
Anti-Mouse IgG (H+L)-HRP Goat, 1 in 10000, Bio-Rad, 1706516

Eukaryotic cell lines

Policy information about cell lines

Cell line source(s)

H9 hESCs (WiCell).
ICSIG-1 IPSC0028 hiPSCs (Sigma).
Wild-type IPSC0028 hiPSCs (Sigma).
Male mouse embryonic fibroblasts (MEFs) isolated from wild-type mouse B6 embryos.
Drosophila S2 cells (ThermoFisher Scientific)

Authentication

All cell lines in this study were authenticated via gene expression analysis.

Mycoplasma contamination

Periodic mycoplasma contamination testing was carried out and confirmed the absence of mycoplasma contamination.

Commonly misidentified lines
(See ICLAC register)

No commonly identified cell lines were used in this study.

Animals and other organisms

Policy information about studies involving animals; ARRIVE guidelines recommended for reporting animal research

Laboratory animals

Wild-type male mouse embryos C57B6/J E14.5. Animals were kept with a maximum of 5 animals per cage and separated by sex.
Illumination was controlled on a 14h light, 10h dark light cycle from 7h to 21h .
Temperature was checked daily and should be 22±2°C
Humidity in mouse rooms was checked daily and should be between 45-70% but can vary with weather conditions, especially in winter.

Wild animals

No wild animals were used in this study.

Field-collected samples

No field-collected samples were used in this study.

Ethics oversight

Animal work carried out in this study was covered by project licences (ECD_P003-2016 and ECD_P170/2019 to V.P and to F.L, respectively) approved by the KU Leuven Animal Ethics Committee.

Note that full information on the approval of the study protocol must also be provided in the manuscript.

ChIP-seq

Data deposition

☒ Confirm that both raw and final processed data have been deposited in a public database such as GEO.

☒ Confirm that you have deposited or provided access to graph files (e.g. BED files) for the called peaks.

Data access links
May remain private before publication.

Calibrated CUT&RUN data provided in GEO (http://www.ncbi.nlm.nih.gov/geo/) under the accession number GSE176175.
http://ftp1.babraham.ac.uk/ftpusr79/

Files in database submission

GSM5569915	Primed_H3K27me3_Replicate_1
GSM5569916	Primed_H3K27me3_Replicate_2
GSM5569917	Primed_UNC1999_H3K27me3_Replicate_1
GSM5569918	Primed_UNC1999_H3K27me3_Replicate_2
GSM5569919	Primed_IgG_Replicate_1
GSM5569920	Primed_IgG_Replicate_2
GSM5569921	Primed_UNC1999_IgG_Replicate_1
GSM5569922	Primed_UNC1999_IgG_Replicate_2
GSM5569923	Naïve_H3K27me3_Replicate_1
GSM5569924	Naïve_H3K27me3_Replicate_2
GSM5569925	Naïve_UNC1999_H3K27me3_Replicate_1
GSM5569926	Naïve_UNC1999_H3K27me3_Replicate_2
GSM5569927	Naïve_IgG_Replicate_1
GSM5569928	Naïve_UNC1999_IgG_Replicate_1
GSM5569929	Naïve_UNC1999_IgG_Replicate_2

Genome browser session
(e.g. UCSC)

http://ftp1.babraham.ac.uk/ftpusr79/Zijlmans_CutandRun_0122_DataHub.json - openable as a remote data track URL in WashU genome browser.

Methodology

Replicates

Two independent biological replicates for all samples except for naive IgG which has one biological replicate. Calibrated CUT&RUN performed for H3K27me3 for experimental samples and IgG, with an anti-Drosophila H2Av spike-in antibody, as a control used for peak calling. Strong replicate agreement between samples of the same condition - Pearson correlation coefficient of 0.76 between two naive H3K27me3 samples and 0.98 for two primed H3K27me3 samples.

Sequencing depth

Total number of sequencing reads: 483,205,175. Highest read count = 53,117,572, Lowest read count = 20,861,009, Average read count = 32,213,678. Uniquely mapping human reads = 275,974,550. Highest uniquely mapping read count = 35,268,030, Lowest uniquely mapping read count = 6,450,381. Average uniquely mapping read count = 18,398,303. Samples were sequenced as 150 bp paired-end sequencing.

Antibodies

Tri-Methyl-Histone H3 Lysine 27 - Cell Signaling Technology, 9733 - (C36B11) Rabbit mAb #9733
Rabbit anti-Mouse IgG (H+L) Secondary Antibody - Invitrogen, 31188 - # 31188
Anti-Histone H2Av spike-in antibody - Active Motif, 61686 - #61686

Peak calling parameters

Peak calling was performed using the CUT&RUN optimised Sparse Enrichment Analysis for CUT&RUN (SEACR) algorithm, using H3K27me3 samples as the sample and their corresponding IgG as a control, against which peaks were called. The top 1% of peaks were retained (v1.3). Peaks closer than 300bp were merged using bedtools merge and peaks common to both replicates were determined by bedtools intersect to generate final peak sets for naive and primed. Peaks called in naive and primed hPSC data sets were concatenated into a combined peak list and deduplicated. Differentially enriched peaks between naive and primed hPSCs were then determined from this concatenated list using a DESEQ2 implementation within SeqMonk (v1.47.2; Babraham Bioinformatics) to identify differential regions with a p-value < 0.05 after Benjamini-Hochberg Multiple-Testing Correction. Common peaks were classified as peaks in the concatenated list that were not statistically-enriched in either condition. These peaks were then filtered against the ENCODE GRCh38 exclusion list to remove coverage outliers.

Data quality

Raw FastQ data were trimmed with Trim Galore and aligned to GRCh38 human genome or Drosophila BDGP6 genome using Bowtie2 with the following parameters –very-sensitive -I 10 -X700. High quality reads with a MAPQ value > 20 were retained by filtering with samtools view. Peak calling was performed using the Sparse Enrichment Analysis for CUT&RUN (SEACR) algorithm and the top 1% peaks were retained. Differentially enriched peaks were determined from a concatenated peak list using a DESEQ2 implementation within SeqMonk to identify differential regions with a p-value < 0.05 after Benjamini-Hochberg Multiple-Testing Correction to ensure a false discovery rate of less than 5%.

Software

Trim Galore (v.0.6.6: Babraham Bioinformatics)
Bowtie2 (v.2.3.2)
samtools (v1.11)
bedtools (v2.29.2)
Sparse Enrichment Analysis for CUT&RUN (SEACR) algorithm (v1.3)
DESEQ2 package within SeqMonk (v1.47.2; Babraham Bioinformatics)
deeptools API suite
Python (v3.7.3)
ChIPseeker package within R (v1.30.3:93)
HOMER
deeptools bamCoverage (v3.43)
UCSC-tools bigWigMerge
USCS-tools bedGraphToBigWig
WashU epigenome browser (v5)
deeptools computeMatrix
deeptools MultiBamSummary
deeptools plotCorrelation
ggplot2 package within R

Flow Cytometry

Plots

Confirm that:

☒ The axis labels state the marker and fluorochrome used (e.g. CD4-FITC).

☒ The axis scales are clearly visible. Include numbers along axes only for bottom left plot of group (a 'group' is an analysis of identical markers).

☒ All plots are contour plots with outliers or pseudocolor plots.

☒ A numerical value for number of cells or percentage (with statistics) is provided.

Methodology

Sample preparation

For Figure 7, blastoids were harvested from the microwell arrays, sequentially treated with 300 U/ml collagenase type-IV and 10x Trypsin-EDTA (ThermoFisher) at 37C on a shaker. The blastoids were dissociated into single cells by pipetting. Cells were washed 3 times with flow buffer (1% FBS in PBS) and incubated with primary antibody diluted in flow buffer and incubated

for 30 minutes at 4°C. The cells were centrifuged, washed with flow buffer twice, and combined with secondary antibody and incubated for 30 minutes at 4°C. The cells were centrifuged, washed twice with flow buffer, and resuspended in fresh flow buffer for flow cytometry analysis.

For Figure 4, naive and primed hPSCs were washed once with PBS and dissociated using Accutase (BioLegend) by incubation for 5 min at 37 °C. Accutase was quenched 1:1 with medium and cells were passed through a 50 µm cell strainer (VWR) and centrifuged at 300 x g for 3 minutes. Cell pellets were washed once with PBS containing 2% FBS (flow buffer) and counted. Fluorophore-conjugated antibodies and eF780 fixable viability dye (eBioscience, 65-0865-14) were mixed with 50 µL Brilliant stain buffer (BD Biosciences) and applied to 500,000 cells in 50 µL flow buffer. Labelling occurred for 30 min at 4 °C in the dark. Cells were washed twice with flow buffer and analysed.

For Figure 5, cells were dissociated using Accutase (5 min incubation at 37°C) and centrifuge at 1000 rpm for 5 min. Supernatant was removed and cell pellets were resuspended in 300 ul of FACS (PBS supplemented with 0,25-0,5% BSA) buffer per sample and centrifuged again in same conditions. Cell pellets were resuspended in 50 ul of FACS buffer and antibody incubations were carried out at 4°C for 30 min. Afterwards, cells were washed twice with 300 ul of FACS buffer and centrifuged (1000 rpm for 5 min). Supernatant was removed and pellet was resuspended in 300 ul of PBS with 4% PFA. Samples were analysed using a BD FACSCanto II Flow Cytometer.

Instrument

BD FACSCanto II Flow Cytometer
BD LSR Fortessa

Software

FlowJo software

Cell population abundance

No cell populations were sorted in this work.

Gating strategy

Single-stained controls were used for compensation calculations and unstained cells were used in cytometer and gating setup.

☒ Tick this box to confirm that a figure exemplifying the gating strategy is provided in the Supplementary Information.
