# Supplementary material for: Integrated multi-omics reveal polycomb repressive complex 2 restricts human trophoblast induction
Source: Nat Cell Biol. 2022 Jun 13;24(6):858–71. doi: 10.1038/s41556-022-00932-w (PMC9203278; doi:10.1038/s41556-022-00932-w)
Supplement: Source Data Extended Data Fig. 4 — Unprocessed western blots and/or gels. [file 41556_2022_932_MOESM16_ESM.pdf]

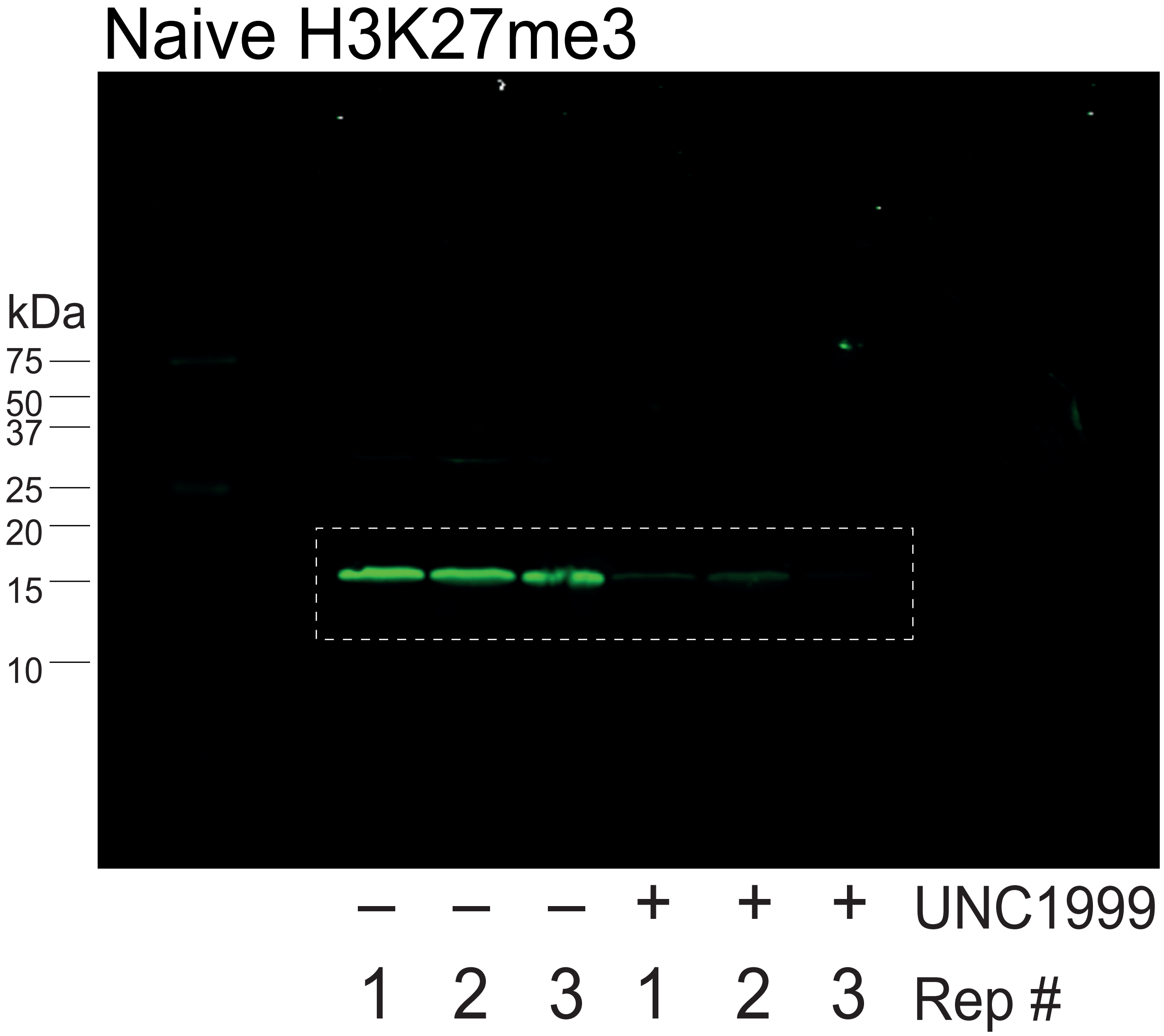

Source Data

Extended Data Figure 4A

Naive H2B loading control

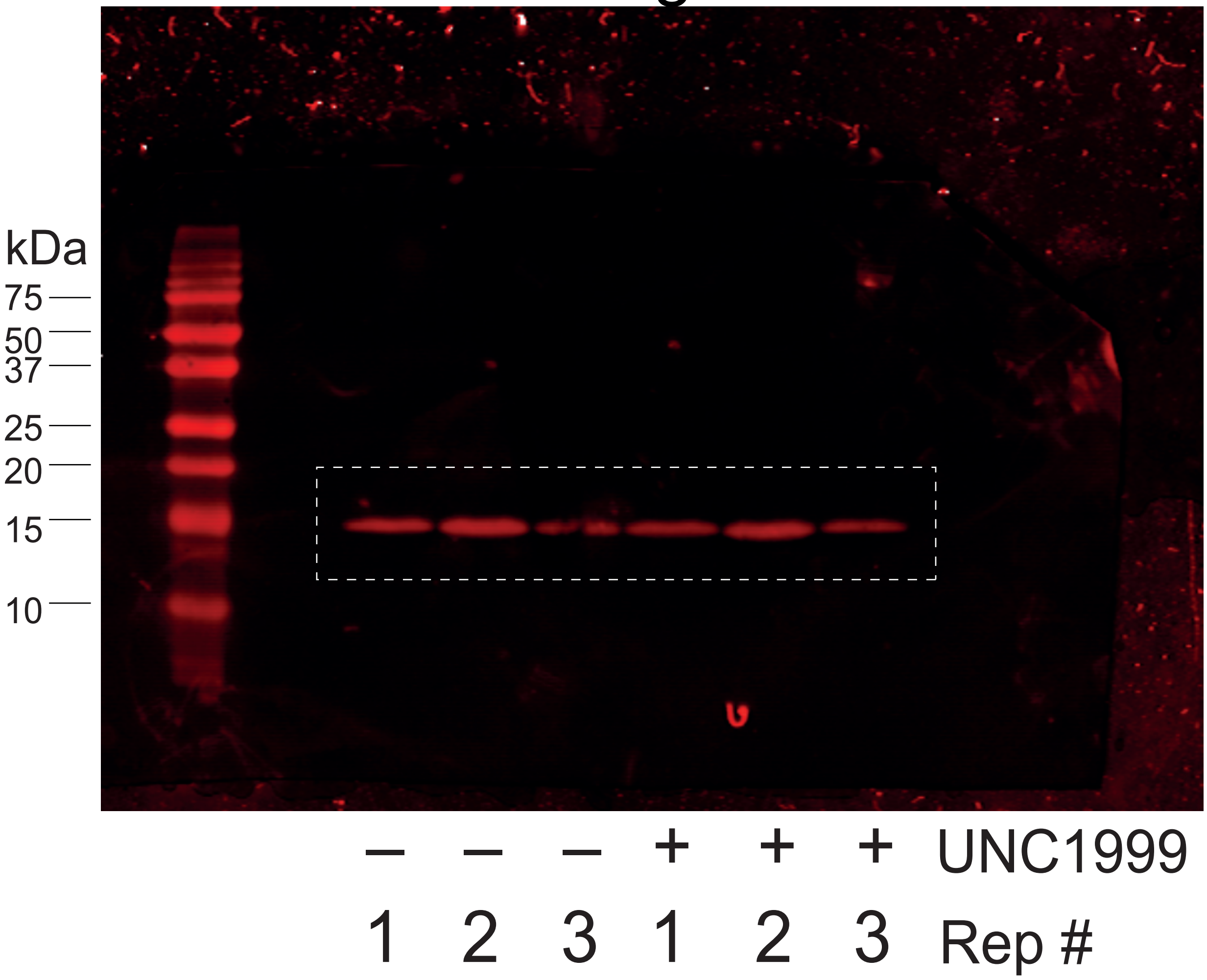

Source Data

Extended Data Figure 4A

Primed H3K27me3

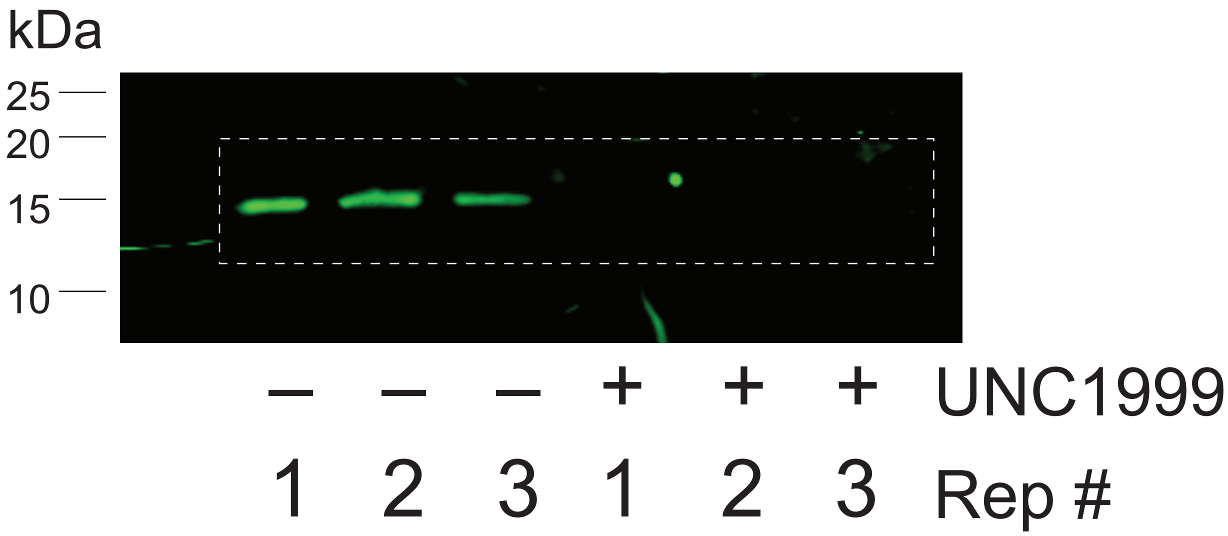

Source Data

Extended Data Figure 4A

Primed H2B loading control

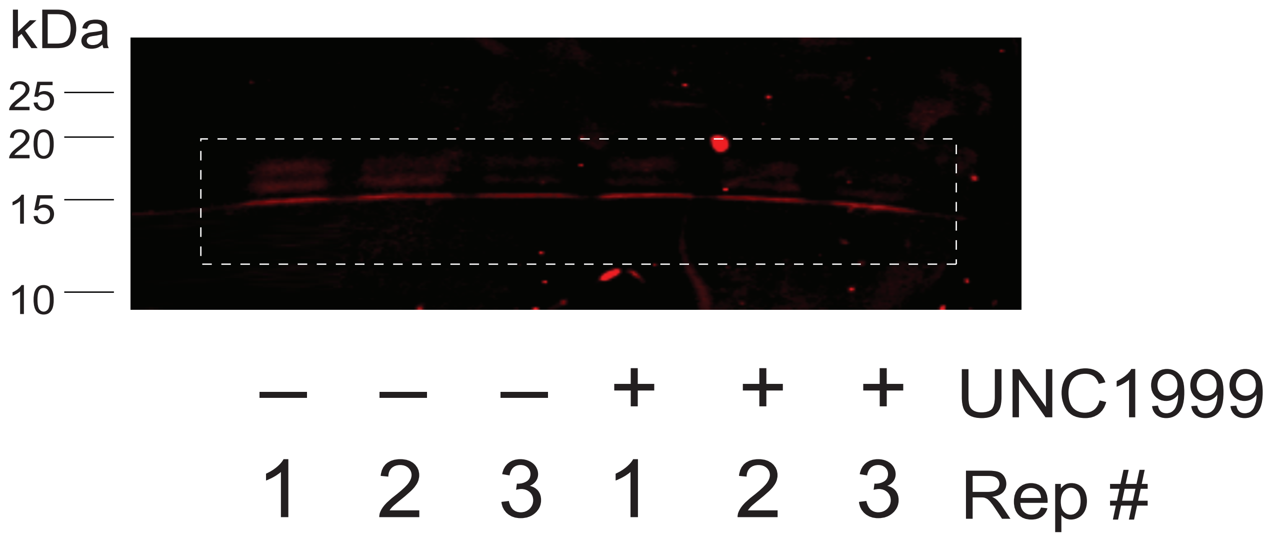

Source Data

Extended Data Figure 4A

Naive H3K27me3

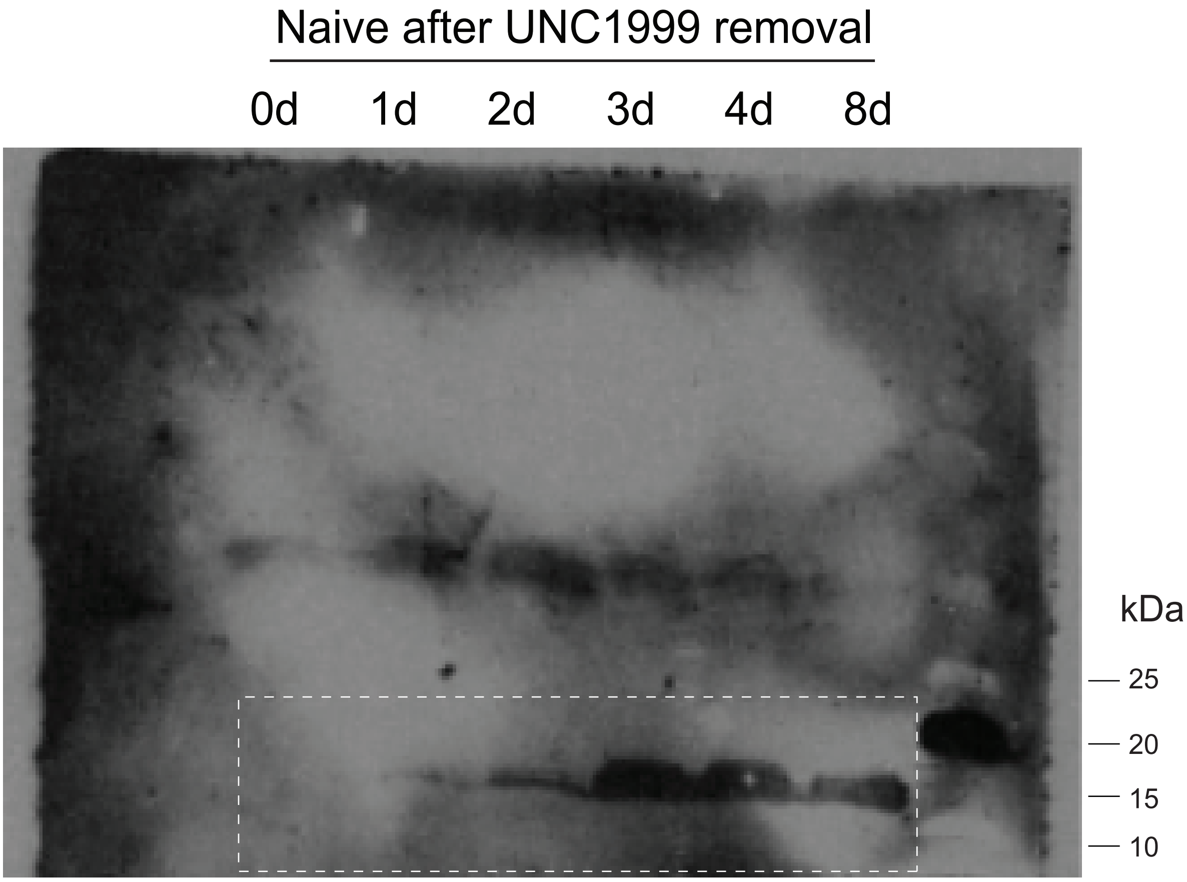

Source Data

Extended Data Figure 4A

Naive H2B loading control

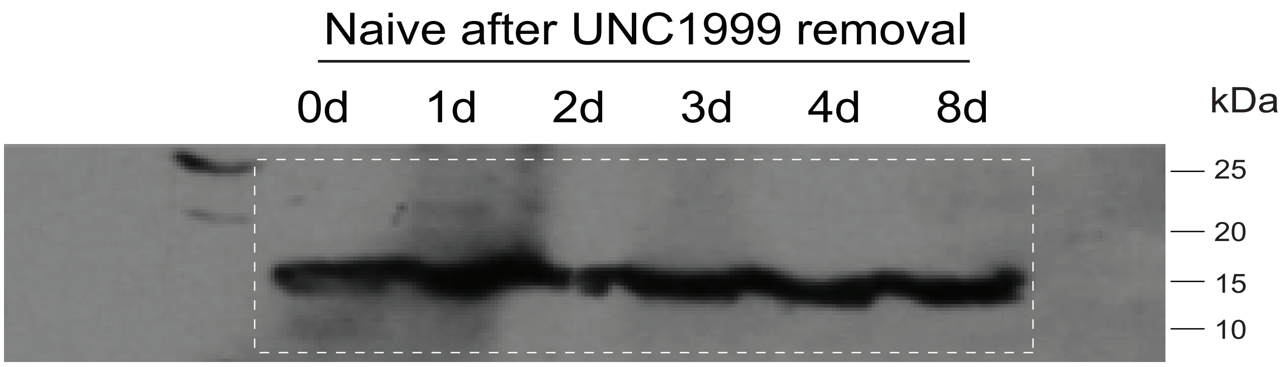

Source Data

Extended Data Figure 4A
